# Synergistic Role of Facet-Engineered Surface and Ferroelectric Polarization in Photoelectrochemical Water Reduction over Pure BiFeO_3_ Thin Film

**DOI:** 10.1021/acsami.5c09048

**Published:** 2025-08-01

**Authors:** Ming-Wei Chu, Yun-Wen Chen, Khian Hooi Chew, Narong Chanlek, Cheng-Sao Chen, Chang Fu Dee, Wei Sea Chang

**Affiliations:** † Mechanical Engineering Discipline, School of Engineering, 175027 Monash University Malaysia, Bandar Sunway, Selangor 47500, Malaysia; ‡ Graduate Institute of Electronics Engineering, and Department of Electrical Engineering, 33561 National Taiwan University, Taipei 10617, Taiwan; § Key Laboratory of Optical Field Manipulation of Zhejiang Province, Department of Physics, 12646 Zhejiang Sci-Tech University, Hangzhou 310018, China; ∇ Zhejiang Expo New Materials Co., Ltd., Wenzhou 325802, China; ∥ Synchrotron Light Research Institute (Public Organization), 111 University Avenue, Muang, Nakhon Ratchasima 30000, Thailand; ⊥ Department of Mechanical Engineering, Hwa Hsia University of Technology, New Taipei City 23567, Taiwan; # Institute of Microengineering and Nanoelectronics (IMEN), Universiti Kebangsaan Malaysia (UKM), Bangi, Selangor 43600, Malaysia; ○ Department of Materials Science and Engineering, National Yang Ming Chiao Tung University, Hsinchu 30010, Taiwan

**Keywords:** BiFeO_3_ thin film, facet engineering, ferroelectric polarization, density functional theory, photoelectrochemical water splitting

## Abstract

Ferroelectric semiconductors have drawn significant attention in photoelectrocatalysis due to their spontaneous ferroelectric polarization, facilitating charge separation and transfer by shielding charge recombination. Nevertheless, the impact of the facet-engineered surface and ferroelectric polarization direction on the polarization strength of a ferroelectric material, surface band bending at the ferroelectrics/electrolyte interface, and charge transport property remains vague. Here, we synthesized p-type BiFeO_3_ (BFO) epitaxial thin films by controlling the (001)-facet and (111)-facet, as well as the upward polarization (P_up_) and downward polarization (P_down_) to systematically investigate ferroelectric polarization-induced internal electric field (*E*_int_) inside BFO and interfacial charge transport mechanism, which in turn affect the overall performance of photoelectrochemical (PEC) water splitting. Our observations demonstrated that the *E*_int_ strength and the charge transport behavior of a BFO can be modulated by facet orientations and polarization directions, leading to huge surface band bending at its BFO/electrolyte interface during PEC reactions. Notably, the BFO film with (111)-facet and P_up_ state showed a ∼5.1-fold enhancement in *E*_int_ compared to the BFO film with (001)-facet and P_down_ state. This was attributed to a ∼1-order reduction in leakage current in (111)-facet-P_up_. These experimental results were further supported by density functional theory (DFT) calculations. Meanwhile, the (111)-facet-P_up_ exhibited a ∼2.8-fold increase in incident photon-to-current efficiency (IPCE) and a ∼4.8-fold improvement in charge transport density, indicating an effective charge separation and reduction in electron–hole recombination with such a synergistic effect. This work emphasizes the synergistic effect of facet-engineered surface and ferroelectric polarization on manipulating charge transfer between ferroelectrics/electrolyte interface and polarization magnitude, offering a generic strategy for optimizing the functionality of ferroelectric-based photoelectrodes for solar-driven water splitting.

## Graphical abstract

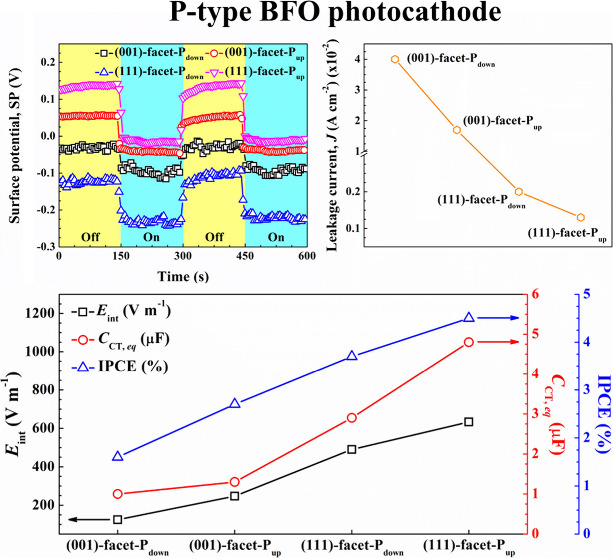

## 1 Introduction

In response to escalating fossil fuel consumption, which has led to the rise of greenhouse gas (GHG), it has become an urgent global priority to boost the rapid transition to sustainable and green energies, particularly clean and renewable hydrogen. The abundant water resources on the planet provide an ideal basis for hydrogen production. Compared to the conventional water electrolysis method, photoelectrochemical (PEC) water splitting has emerged as the eco-friendly and promising strategy for producing “green” hydrogen as its solar-to-chemical conversion process generates negligible environmental pollution.^1–5^ However, the development of an optimal photoelectrode for PEC activities can be challenging since the performance of the photoelectrode fundamentally depends on three criteria: (i) favorable light harvesting; (ii) efficient charge separation and transfer; and (iii) suitable catalytic reaction at the solid/electrolyte interface. Among these criteria, effective charge separation and transport play an important role in PEC reactions because they strongly determine the success of water reduction (H^+^/H_2_) or oxidation (O_2_/H_2_O).

Ferroelectric semiconductors, such as KNbO_3_ (KNO),^6^ BiFeO_3_ (BFO),^7–13^ BaTiO_3_ (BTO),^14–17^ PbTiO_3_ (PTO),^18–22^ Bi_4_NbO_8_Cl (BNOC),^23^ and Bi_2_FeCrO_6_ (BFCO),^24^ have emerged as promising photoelectrodes or photocatalysts for water splitting. Their potential arises from intrinsic ferroelectric polarization, which induces an internal electric field. The presence of an internal electric field not only facilitates charge separation and transfer by inhibiting charge recombination but also modifies surface chemistries via polarization switching.^25–27^ In ferroelectric materials, switchable polarization can be either upward polarization or downward polarization, corresponding to the polarization vector being towards or away from the ferroelectric material’s surface. It additionally can manipulate charge dynamics and surface band bending at the ferroelectrics/electrolyte interface, thereby tuning water redox activities. Among conventional ferroelectric perovskites, BFO stands out owing to lead-free nature, making it more environmentally friendly compared to PTO. Additionally, BFO possesses a relatively narrow bandgap of 2.1–2.7 eV,^28,29^ which is smaller than the wide bandgap of 3.0–4.0 eV in KNO,^6^ BTO,^14^ and PTO.^18^ This narrow bandgap enables BFO to effectively generate electron–hole pairs under the visible-light spectrum. Meanwhile, BFO exhibits a large spontaneous polarization of 110 μC cm^–2^,^30^ which aids in accelerating charge separation and migration efficiency upon illumination. These benefits collectively make BFO a highly attractive photoelectrode candidate for PEC water splitting. In parallel, recent studies have reported promising optoelectronic properties in two-dimensional (2D) perovskite oxides,^51–55^ opening new avenues for enhancing light–matter interaction and charge dynamics. These insights support the continuous development of BFO-based systems and similar materials for advanced PEC applications.

The impact of ferroelectric polarization on surface band bending and charge dynamics has been widely studied across various ferroelectric nanostructures. However, the reported polarization vector was typically aligned along the [001] crystallographic direction, for example, BNOC {001} nanosheet,^23^ KNO (001) nanowire,^6^ BFO (001) nanosheet,^13^ BTO (001) nanoparticle,^16^ and PTO {001} nanoplate.^18–21^ In these ferroelectric photocatalysts, photoinduced electrons and holes tend to migrate towards upward-poled (001) and downward-poled (001) surfaces, respectively. Abbasi et al.^17^ used density functional theory (DFT) calculations to reveal that the upward polarized BTO (001) surface exhibits better photocatalytic (PC) activities than the downward polarized counterpart. Huang et al.^11^ systematically compared the spontaneous polarization along the (001), (110), and (111) orientations in the p-type BFO epitaxial thin films. The authors demonstrated that upward polarization along the (111) orientation significantly improves the PEC water reduction. In contrast, in the work of n-type BFO thin films reported by Song et al.,^10^ the downward polarization along the (111) orientation largely enhanced the PEC water oxidation. Both the experimental studies pointed out the role of facet orientation in determining the strength of ferroelectric polarization-induced internal electric field, thereby governing charge dynamics and surface band bending at the BFO/electrolyte interface during PEC water splitting. Nonetheless, the underlying mechanisms linking facet orientation to the ferroelectric polarization magnitude and charge transfer efficiency remain unclear.

In this work, BFO was chosen as a model material to unveil the effect of the facet-engineered surface and polarization direction on charge transport efficiency and ferroelectric polarization-induced internal electric field (*E*_int_) strength, in which both have strong influence on the overall performance of PEC water splitting. A series of p-type BFO epitaxial thin films with controlled facet orientations and spontaneous polarization directions were fabricated by the pulsed laser deposition (PLD) technique. Our prepared BFO thin films were identified as (001)-facet-P_down_, (001)-facet-P_up_, (111)-facet-P_down_, and (111)-facet-P_up_. Among these films, the (111)-facet-P_up_ was found to exhibit the highest *E*_int_ magnitude and charge transfer efficiency coupled with the lowest leakage current. These experimental observations were further supported by DFT computations. As a result, the (111)-facet-P_up_ possessed the largest charge separation and transportation, resulting in huge charge accumulation and a downward band bending degree at the BFO/electrolyte interface that provides effective PEC water reduction.

## 2 Experimental Section

### 2.1 Growth of BFO Thin Film Heterostructures

The BFO thin films were epitaxially deposited on the La_0.7_Sr_0.3_MnO_3_ (LSMO)-buffered SrTiO_3_ (STO) (001) and (111) single-crystal substrates by employing pulsed laser deposition (PLD-12-Super, AdNaNotek Corp.) with a 248 nm KrF excimer laser (Coherent COMPexPro 102 F). The laser energy was 180 mJ with a repetition rate of 10 Hz. The LSMO buffer layer was grown on the STO substrate at 700 °C in an O_2_ pressure of 200 mTorr. The BFO film was subsequently grown on top of the LSMO layer at 700 °C in an O_2_ pressure of 200 mTorr. Thereafter, the BFO/LSMO samples were gradually cooled to room temperature at 10 °C min^–1^ at the same O_2_ pressure. Similarly, the BFO thin films were deposited on the SrRuO_3_ (SRO)-buffered STO (001) and (111) single-crystal substrates using the same deposition parameters as the above.

### 2.2 Material Characterizations

The phase identification was conducted using X-ray diffraction (XRD, Bruker D8 Discover X-ray Diffractometer) in θ–2θ scanning range from 10° to 80° with Cu Kα radiation (λ = 1.5406 Å). The plane views of as-prepared BFO thin films were probed by a field emission scanning electron microscope (FESEM, JEOL JSM-7610 F Plus) at an accelerating voltage of 15 kV. High-resolution transmission electron microscopy (HRTEM, FEI TECNAI G2 F20 S-TWIN) was used to determine the thickness of thin films and to obtain the selected area electron diffraction patterns (SAED) at an accelerating voltage of 200 kV. The HRTEM specimens were prepared by a dual-beam focused ion beam (FIB, SEIKO SMI3050SE) with a Ga ion source under an accelerating voltage of 30 kV. A 500 nm thick carbon (C) layer was deposited on the sample surface to protect the specimen during polishing. The UV–Vis absorption spectra were recorded at room temperature by using UV–Vis absorption spectroscopy (Cary 100 UV–Vis Spectrophotometer, Agilent). The valence band maximum of the BFO sample was determined using high-resolution X-ray photoelectron spectroscopy (HR-XPS, PHI5000 Versa Probe II, ULVAC-PHI, Japan) equipped with the Al Kα X-ray source of 1486.6 eV. Photoluminescence (PL) spectra were acquired using Labram HR Evolution, Horiba Scientific with a 325 nm light excitation. Time-resolved photoluminescence (TRPL) spectra were obtained via the fluorescence lifetime system (DeltaPro, Horiba Scientific) with a light excitation wavelength of 317 nm.

### 2.3 Electrical Measurements

Piezoresponse force microscopy (PFM) and Kelvin probe force microscopy (KPFM) measurements were conducted using a Bruker MultiMode 8 atomic force microscope (AFM) with a conductive platinum-coated cantilever tip (OMCL-AC240TM-B3, resonant frequency 70 kHz, spring constant 2 N m^–1^). The out-of-plane (OOP) PFM phase result was obtained in contact mode with a scan rate of 0.5 Hz and a driving frequency range of 247–252 kHz, which closely matched the contact resonance frequency (*f*_0_) of 255 kHz to optimize the signal-to-noise ratio (SNR). The driving amplitude of the AC bias range was maintained at 200–400 mV. The PFM response was examined both before and after applying the DC biases of +6 and –6 V to the tip. For the KPFM measurement, PeakForce Tapping mode was employed at a scan rate of 0.5 Hz with an approximate lift scan height of 98 nm. A DC bias is routed to the tip to nullify the bias between the tip and the sample surface. The surface potential (SP) is collected in the dark and under 405 nm illumination. Light-induced change in SP can be expressed as follows:^31^

1 $$\mathrm{SPD}={\mathrm{SP}}_{\mathrm{light}}-{\mathrm{SP}}_{\mathrm{dark}}$$

where the SP_light_ and SP_dark_ are the surface potentials measured under illumination and in the dark, respectively. Here, the surface potential difference (SPD) is also known as the surface photovoltage (SPV). The leakage current density–voltage (*J*–*V*) curves and polarization–coercive voltage (*P*–*V*) hysteresis loops of the films were obtained at room temperature and at applied voltage ranging from −10 V (−166 V cm^–1^) to +10 V (+166 V cm^–1^) using a Precision multiferroic II ferroelectric tester (Radiant Technologies). The leakage current density for each BFO sample was obtained by averaging the results from three independent measurements. During the leakage current and ferroelectric polarization measurements, the probe was aligned perpendicular to the film plane, with the buffer layer (LSMO or SRO) acting as the bottom electrode and a 30 nm thick silver (Ag) serving as the top electrode.

### 2.4 Photoelectrochemical Examinations

The PEC examinations, including linear sweep voltammetry (LSV), 4 h chronoamperometry (CA) stability, electrochemical impedance spectroscopy (EIS) Nyquist curve, and Mott–Schottky (MS) plot, were conducted at room temperature using a Gamry Interface 1000E potentiostat. A standard three-electrode configuration was employed, with a BFO thin film heterostructure acting as the working electrode, a platinum (Pt) as the counter electrode, and Ag/AgCl (saturated with 3.0 M KCl) as the reference electrode. All the electrodes were immersed in a 0.5 M sodium sulfate (Na_2_SO_4_) electrolyte (pH = 7.0). The potential was vs. Ag/AgCl in aqueous media. The light source was the AM1.5G simulated solar light (100 mW cm^–2^) integrated with a Xe arc lamp (CHF-XM-500W). The incident photon-to-current efficiency (IPCE) was examined under monochromatic light in the wavelength range of 300–600 nm, using a Xenon lamp coupled with a monochromator (Newport, TLS130B-300X).

### 2.5 Theoretical Modeling

The structures of BFO surfaces were simulated using the Vienna Ab initio Simulation Package (VASP) at the level of density functional theory (DFT).^32,33^ The BFO slabs were constructed by stacking rhombohedral (*R*3*c*) BFO in the conventional [001] and [111] directions. The optimized lattice constants of *R*3*c* BFO were *a* = *b* = *c* = 5.66 Å and a *R*3*c* angle of *a* = 59.2°.^34^ A BFO [001] slab model consisted of 6 atomic layers with 2 × 2 lateral cross sections, and the bottom four layers were fixed. On the other hand, a BFO [111] slab model contained eight atomic layers with 2 × 2 lateral cross-section and the bottom five layers fixed. The other BFO surface atoms are free to move in structural optimization. A vacuum layer of 15 Å was inserted into both the BFO [001] and [111] slab models to prevent a strong interaction between the top and bottom BFO surfaces. For a better projected density of states (PDOS) for BFO surfaces, an additional Hubbard-U = 3 eV was applied to Fe d orbitals in all calculations to increase the underestimated bandgap of BFO to 1.97 eV in DFT.^34^ The spin polarized wave functions of Bi (6s6p5d), Fe (4s3p3d), and O (2s2p) atoms were expanded using a plane wave basis set with an energy cutoff of 500 eV, in conjunction with pseudopotentials constructed by the projected augmented wave method.^35^ A G-type antiferromagnetic (AFM) arrangement on the Fe sites was carefully assigned for all simulations. Perdew–Burke–Ernzerhof generalized gradient approximation (PBE-GGA) functionals were used to obtain electron exchange-correlation energies.^36^ A 5 × 5 × 1 Γ-centered Monkhorst–Pack k-point mesh was applied for structural optimization.^37^ All the structures were optimized until the force on each atom was less than 0.01 eV/Å. The electron local potential profiles of all the BFO slabs along the *z*-axis were calculated by averaging the lateral cross-section of the three-dimensional local potential.

## 3 Results and Discussion

### 3.1 Material Characterizations

XRD was employed to characterize the crystalline structures of BFO/SRO (001), BFO/LSMO (001), BFO/SRO (111), and BFO/LSMO (111), as shown in Figure 1. The XRD θ–2θ measurement in Figures 1a and 1b reveals the diffraction peaks of BFO thin films located at 2θ degree of ∼45.54°, suggesting the (001)-facet perovskite structure with respect to the STO (001) diffraction peak. Simultaneously, Figures 1c and 1d depict the peaks of both the BFO films positioned at a 2θ degree of ∼38.90°, indicative of the (111)-facet perovskite structure for the STO (111) peak. The wide XRD θ–2θ profiles ranging from 10° to 80° (Figure S1) show the same crystallographic orientations as the corresponding STO substrates. The FESEM plane views of all of the samples are shown in Figure S2.

**Figure 1:**
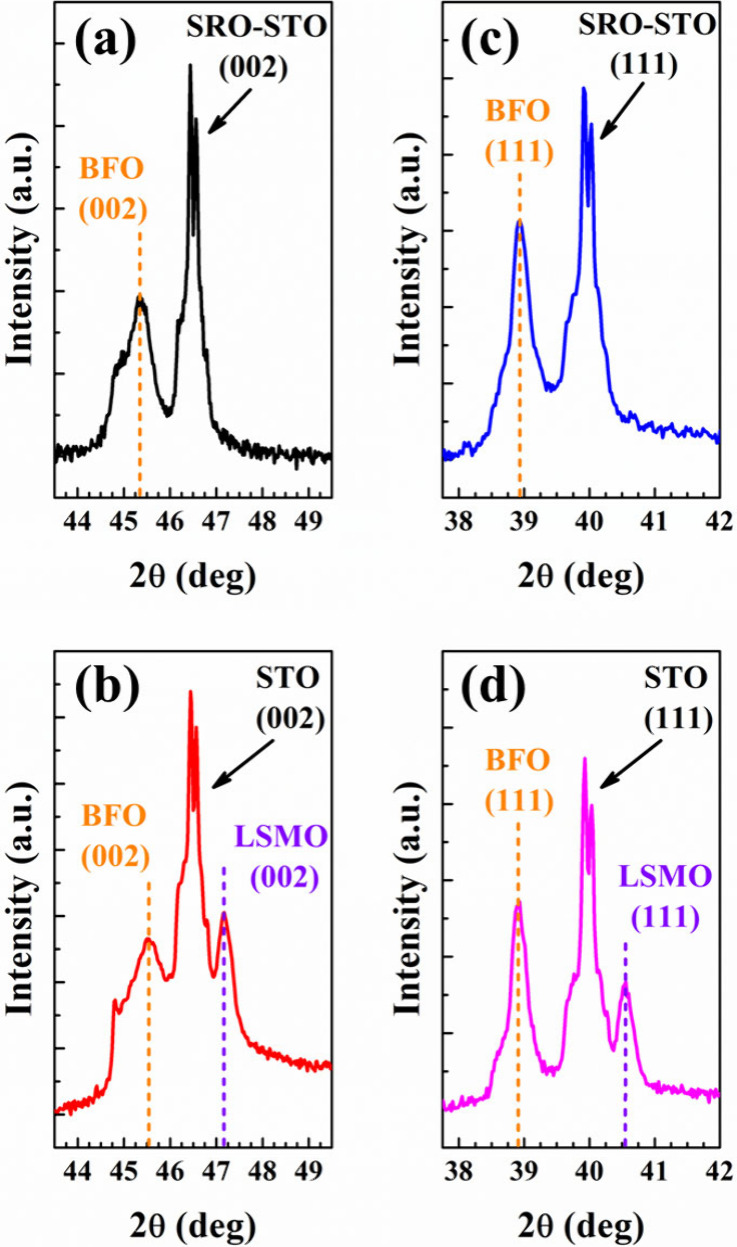
XRD patterns of (a) BFO/SRO (001), (b) BFO/LSMO (001), (c) BFO/SRO (111), and (d) BFO/LSMO (111).

Figure 2a displays the optical absorption spectra of each BFO film, with the onset absorption observed in the wavelength range of 450–550 nm, suggesting that all the BFO samples are photosensitive to the visible-light spectrum and favorable for solar-driven water splitting under AM1.5G illumination. Meanwhile, the optical bandgap energy (*E*_g_) can be estimated using Tauc’s relation:^38^

2 $${\left(\alpha hv\right)}^{n}=A\left(hv-E_{g}\right)$$

where α is the absorption coefficient, *hv* is the photon energy, *n* = 2 denotes the power index based on the direct energy transition, *A* is the scaling constant, and *E*_g_ symbolizes the direct optical bandgap. By extrapolation of the slopes in Figure 2b, the *E*_g_ values of BFO/SRO (001), BFO/LSMO (001), BFO/SRO (111), and BFO/LSMO (111) are calculated to be 2.62, 2.58, 2.53, and 2.46 eV, respectively. XPS was used to explore the valence band maximum (VBM) of BFO/LSMO (111) with respect to the Fermi level (*E*_F_), in which the *E*_F_ is 0.4 eV away from the VBM, as shown in Figure S3. This indicates that all of the BFO films generally illustrate a p-type semiconductor characteristic.

**Figure 2:**
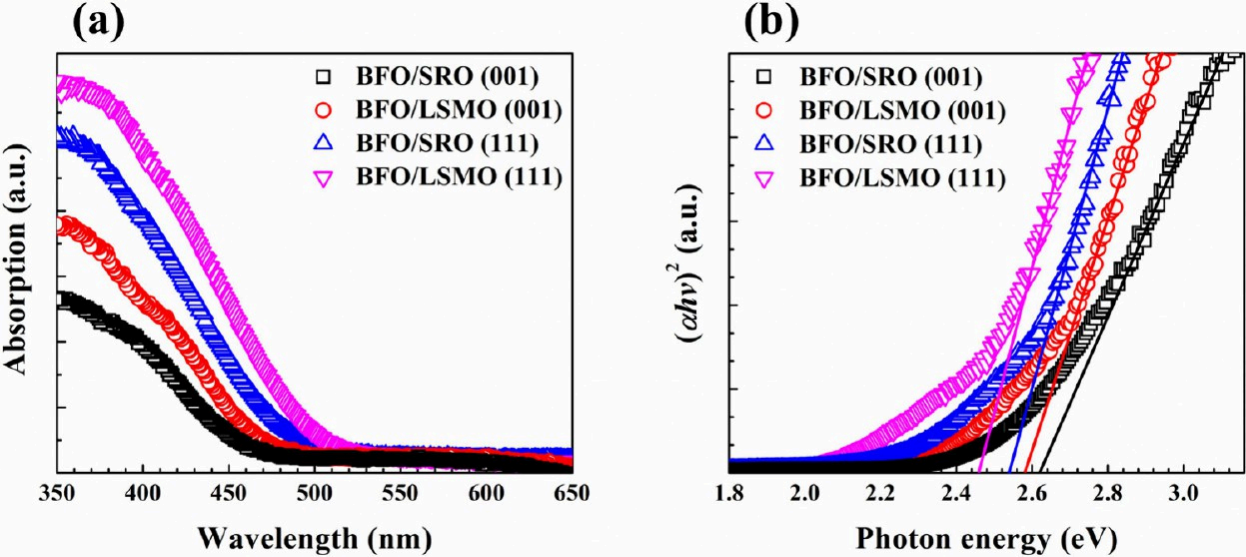
(a) UV–Vis absorption spectra and (b) Tauc’s plots of BFO/SRO (001), BFO/LSMO (001), BFO/SRO (111), and BFO/LSMO (111).

Figures 3a and 3b present the cross-sectional TEM images of the BFO thin films deposited on LSMO-buffered STO (001) and (111) substrates, respectively. In both the BFO (001) and (111) samples, the LSMO layers have a thickness of 15 nm, while the BFO films are 60 nm thick. The selected area electron diffraction (SAED) patterns of the BFO (001)-facet and BFO (111)-facet were obtained along the [010] and [011] zone axes, as depicted in Figures 3c and 3d, respectively. The SAED results reveal that the BFO (001)-facet shows a (001)-oriented rhombohedral (*R*3*c*) symmetry, while a (111)-oriented *R*3*c* symmetry is exhibited in the BFO (111)-facet.^39^ These structural findings are in excellent agreement with the XRD results.

**Figure 3:**
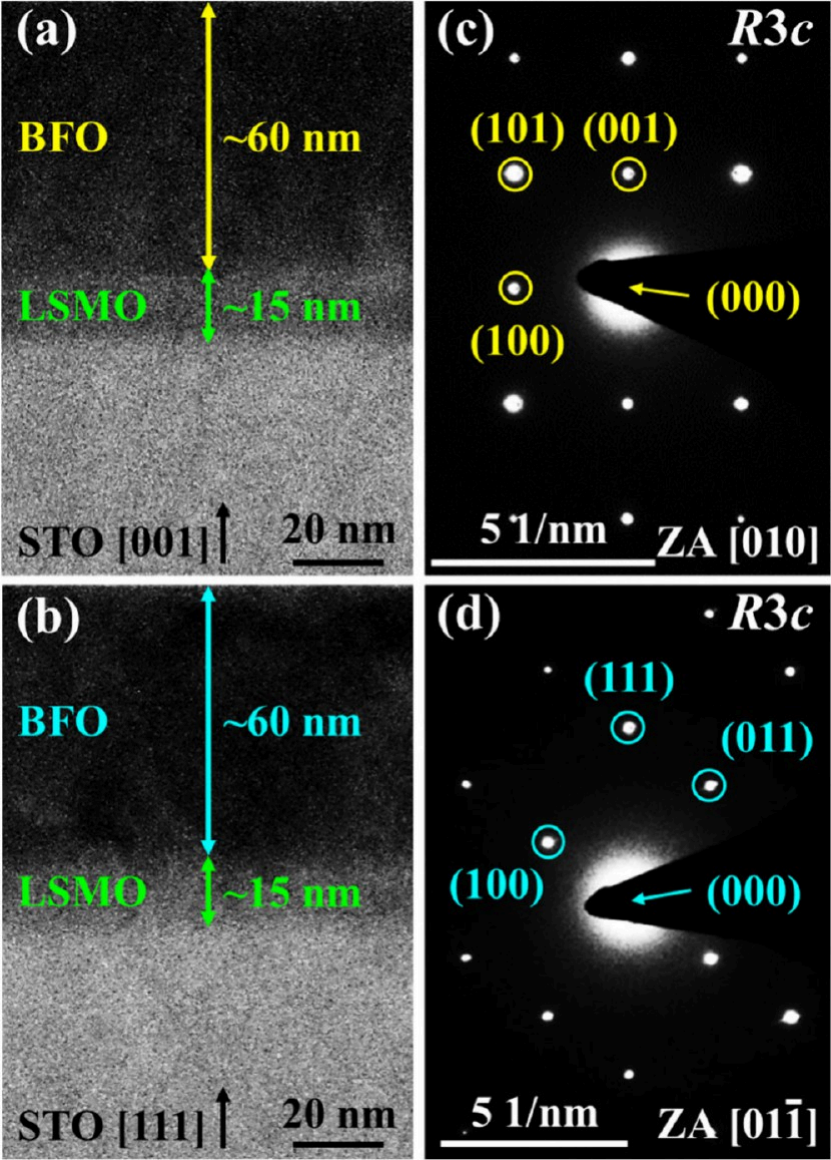
Cross-sectional TEM images of (a) BFO/LSMO (001) and (b) BFO/LSMO (111). SAED patterns of BFO films in the (c) (001)-facet and (d) (111)-facet along with the [010] and [011] zone axes, respectively.

Out-of-plane (OOP) PFM was used to study the polarization directions of the BFO/SRO (111) (Figure 4a) and BFO/LSMO (111) (Figure 4c). In the absence of an applied bias (0 V), the PFM of BFO/SRO (111) reveal a distinct P_down_ with the presence of dark region, while the BFO/LSMO (111) exhibits a clear P_up_ with bright region. Moreover, the PFM phase hysteresis loops of BFO/SRO (111) (Figure S4a) and BFO/LSMO (111) (Figure S4b) shifting toward the negative and positive bias regions indicate the preferential P_down_ and P_up_ states, respectively. The work published by Yu et al.^40^ demonstrated that the as-grown P_down_ (P_up_) direction in the BFO/SRO (BFO/LSMO) heterostructure is the resultant of the interfacial valence mismatch between SRO and BFO (LSMO and BFO) interface. Thus, the precise control of spontaneous polarization direction in each BFO sample confirms the high-quality epitaxial deposition of PLD. Besides, polarization switching in the 2 × 2 μm^2^ box region is successfully achieved by applying an external DC bias to the BFO/SRO (111) and BFO/LSMO (111), which illustrates a clear ∼180° phase degree contrast in Figures 4b and 4d, respectively. For example, as-grown P_down_ in BFO/SRO (111) is switched to P_up_ when a DC bias of −6 V is applied and subsequently rebounds to P_down_ under a bias of +6 V. In the case of BFO/LSMO (111), inherent P_up_ transitions to P_down_ under a DC bias of +6 V and then reverts to P_up_ under a bias of −6 V. Based on the PFM results, our prepared BFO/SRO (001), BFO/LSMO (001), BFO/SRO (111), and BFO/LSMO (111) can be hereafter denoted as (001)-facet-P_down_, (001)-facet-P_up_, (111)-facet-P_down_, and (111)-facet-P_up_, respectively.

**Figure 4:**
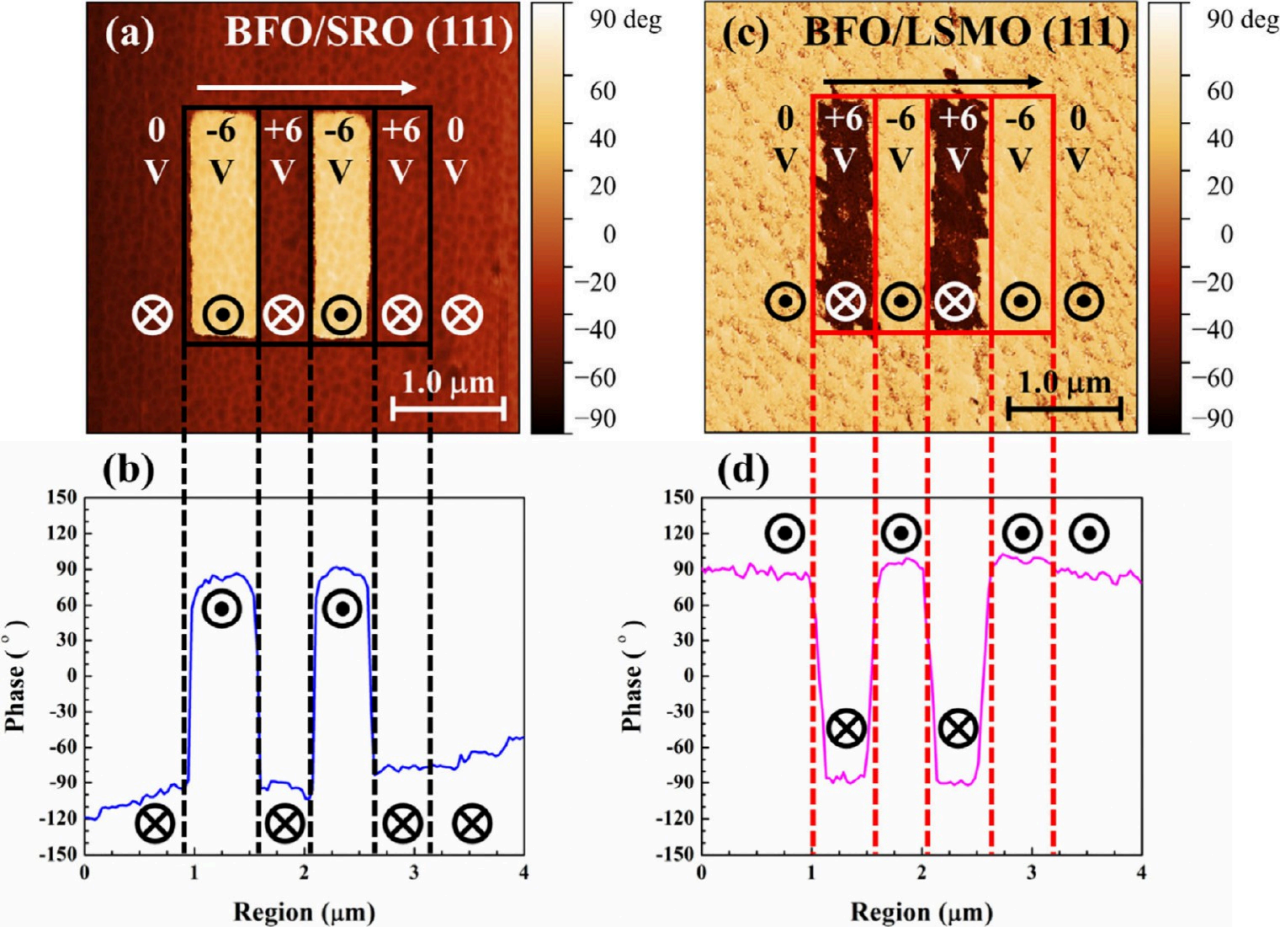
OOP PFM phase images and corresponding phase degree profiles of (a, b) BFO/SRO (111) and (c, d) BFO/LSMO (111). The arrow indicates the poling sequence. The symbols of ⊗ and ⊙ suggest the downward and upward polarizations, respectively.

### 3.2 Electrical and Ferroelectric Characteristics

To investigate the effect of facet orientation and polarization direction on electrical conductivity, the room temperature leakage current density–voltage (*J*–*V*) curves of (001)-facet-P_down_, (001)-facet-P_up_, (111)-facet-P_down_, and (111)-facet-P_up_ were carried out, as exhibited in Figure 5a. The leakage current density (*J*) of each BFO sample gradually increases with an increase in applied voltage. Among these samples, the (111)-facet-P_up_ generally shows a ∼1-order reduction in *J* compared to the (001)-facet-P_down_. This is also evident at an applied voltage of 1 V (Figure 5b), where the (111)-facet-P_up_ reveals the *J* of 1.3 × 10^–3^ A cm^–2^, significantly lower than that of 4.0 × 10^–2^ A cm^–2^ in the (001)-facet-P_down_. These leakage results suggest that the BFO (111)-facet generally gives better electrical conduction. Interestingly, the *J* response is not only dependent on the facet orientation, but also on the direction of polarization (P_down_ or P_up_). Leakage current in BFO can be affected by defect states, interface band alignment, and charge carrier injection barriers.^41,57,58^ Among these, oxygen vacancies (common intrinsic defects in BFO) play a pivotal role.^59,60^ Meanwhile, these oxygen vacancies can be redistributed by polarization direction.^41,61,62^ Under upward polarization, electrostatic forces drive these oxygen vacancies toward the bottom electrode, effectively depleting the surface region of defect states. This redistribution mitigates defect-assisted leakage pathways and reduces surface recombination losses. In contrast, downward polarization induces the migration of oxygen vacancies toward the BFO surface, thereby increasing the defect density at the BFO/electrode interface. These vacancies act as trap states, facilitating tunneling-assisted leakage currents and enhancing recombination losses. This could explain why the upward-polarized BFO films generally show the lower *J* response compared to the downward-polarized BFO films in this work.

**Figure 5:**
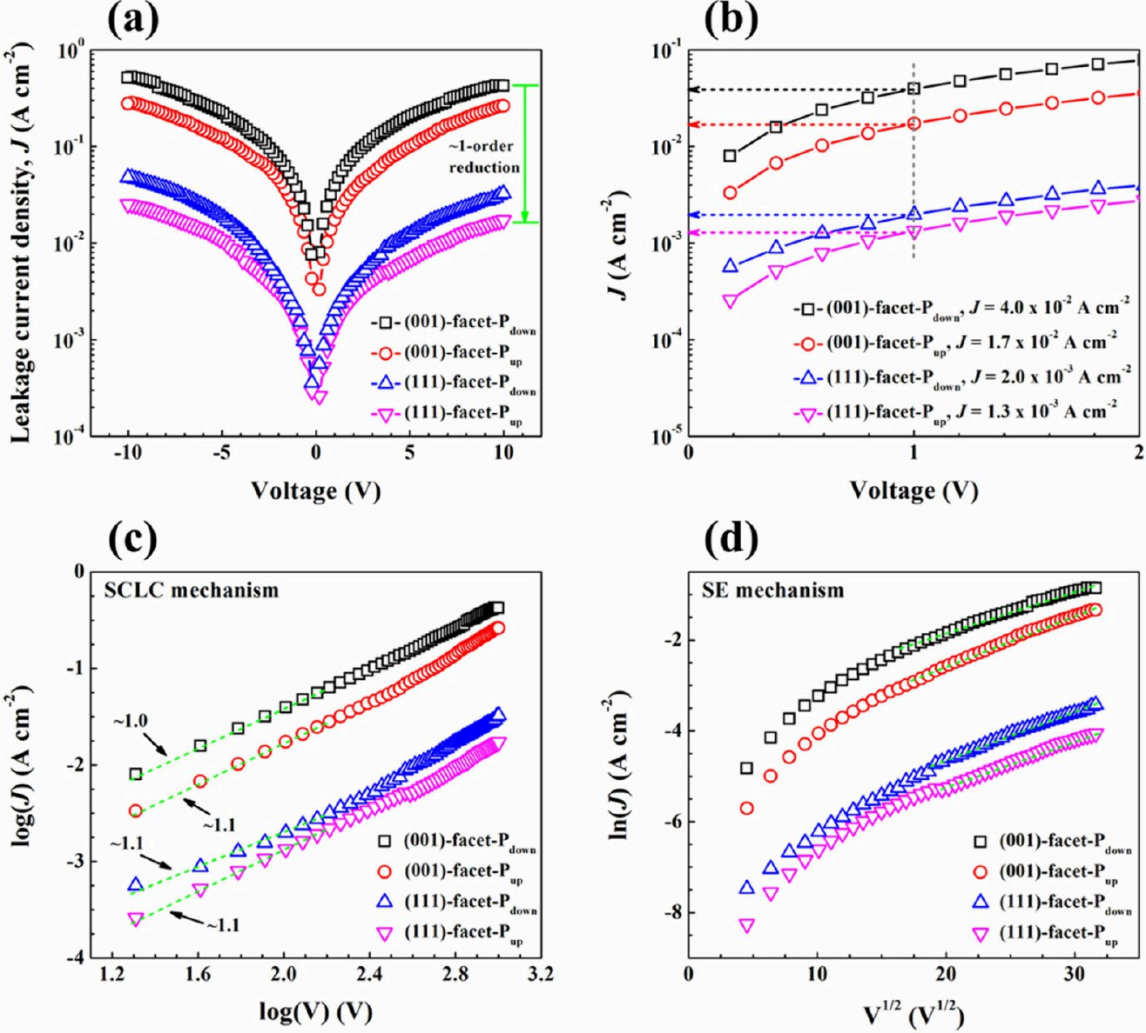
(a) Leakage current density–voltage (*J*–*V*) curves of (001)-facet-P_down_, (001)-facet-P_up_, (111)-facet-P_down_, and (111)-facet-P_up_. (b) Narrow *J*–*V* curves of the corresponding samples at applied voltage ranging from 0 V to 2 V. Leakage current mechanisms presented as (c) SCLC and (d) SE models for all the samples.

To further explore the leakage behavior of all the BFO samples, the space-charge-limited conduction (SCLC), Poole–Frenkel (PF) emission, Fowler–Nordheim (FN) tunneling, and Schottky emission (SE) are considered.^60,63^ Among them, the FN mechanism is excluded as the thickness of as-prepared BFO films is 60 nm, which is larger than 3 nm for the tunneling depth.^64^ Also, the PF mechanism can be ruled out, since the corresponding plots (*V* ^1/2^–ln *J*/*V*) shown in Figure S5 do not show any clear linear behavior. Therefore, the SCLC and SE mechanisms are suitable for exploring the leakage behavior of all the films. The mechanism of SCLC originates when the rate of charge injection from the electrode to the film exceeds the rate at which charges can travel through the film, leading to space charges. The SCLC mechanism can be expressed as:

3 $$J_{\mathrm{SCLC}}=\left(9{\mu \varepsilon }_{r}\varepsilon_{0}V^{2}\right)/\left(8L\right)$$

where μ is the charge mobility, ε_r_ represents the relative dielectric constant, ε_0_ indicates the permittivity of free space, *V* is the voltage, and *L* is the thickness of the film. The mechanism of SE arises from the difference in Fermi levels between the electrode and the film. The energy difference gives rise to a potential energy barrier at the interface that charge carriers must overcome. The current density across the Schottky barrier is presented by the following equation:

4 $$J_{\mathrm{SE}}={AT}^{2}\exp\left(\left(\Phi /k_{B}T\right)-\left(1/k_{B}T\right){\left(\left(q^{3}V\right)/\left(4{\pi \varepsilon }_{r}\varepsilon_{0}L\right)\right)}^{1/2}\right)$$

where *A* is the Richardson constant and Φ is the height of the Schottky barrier. The SCLC and SE mechanisms are analyzed using the respective plots: log *V*–log *J* for SCLC (eq 3) and log *V* ^1/2^–ln *J* for SE (eq 4). After the fitting, a clear linear relationship in the log *V*–log *J* plot (Figure 5c) is observed in the low voltage region with the corresponding slopes of ∼1 for all the films, indicating Ohmic conduction. In contrast, the *V* ^1/2^–ln *J* curve (Figure 5d) exhibits linearity at the high voltage region, consistent with the Schottky emission.

The ferroelectric polarizations of the (001)-facet-P_down_, (001)-facet-P_up_, (111)-facet-P_down_, and (111)-facet-P_up_ were investigated by the polarization–coercive voltage (*P*–*V*) hysteresis loops (Figure 6a). Generally, the BFO (111)-facet with a rhombohedral structure possesses a remarkable ferroelectric polarization compared to the BFO (001)-facet owing to the large displacement of the bismuth (Bi) cations relative to the oxygen (O) octahedral along the out-of-plane [111] direction.^42^ The remnant polarization (*P*_r_) presented in Figure 6b confirms that the BFO (111)-facets have the larger *P*_r_ values compared to the BFO (001)-facets, which is in the same trend as the reported works.^42–44^ Among all the BFO samples, the *P*_r_ follows the order (111)-facet-P_up_ (37 μC cm^–2^) > (111)-facet-P_down_ (31 μC cm^–2^) > (001)-facet-P_up_ (22 μC cm^–2^) > (001)-facet-P_down_ (14 μC cm^–2^). The (111)-facet-P_up_ outperforms all other orientations in terms of ferroelectric polarization. The higher *P*_r_ value implies that this configuration facilitates a stronger dipole alignment, which can lead to enhanced internal electric fields (*E*_int_). Moreover, it was found that the BFO film in the P_up_ state exhibits a higher *P*_r_ than the P_down_ state. The P_up_ state is associated with lower leakage current densities, which likely prevent charge carriers from dissipating prematurely. This reduction in the leakage current contributes to maintaining a higher *P*_r_ value.

**Figure 6:**
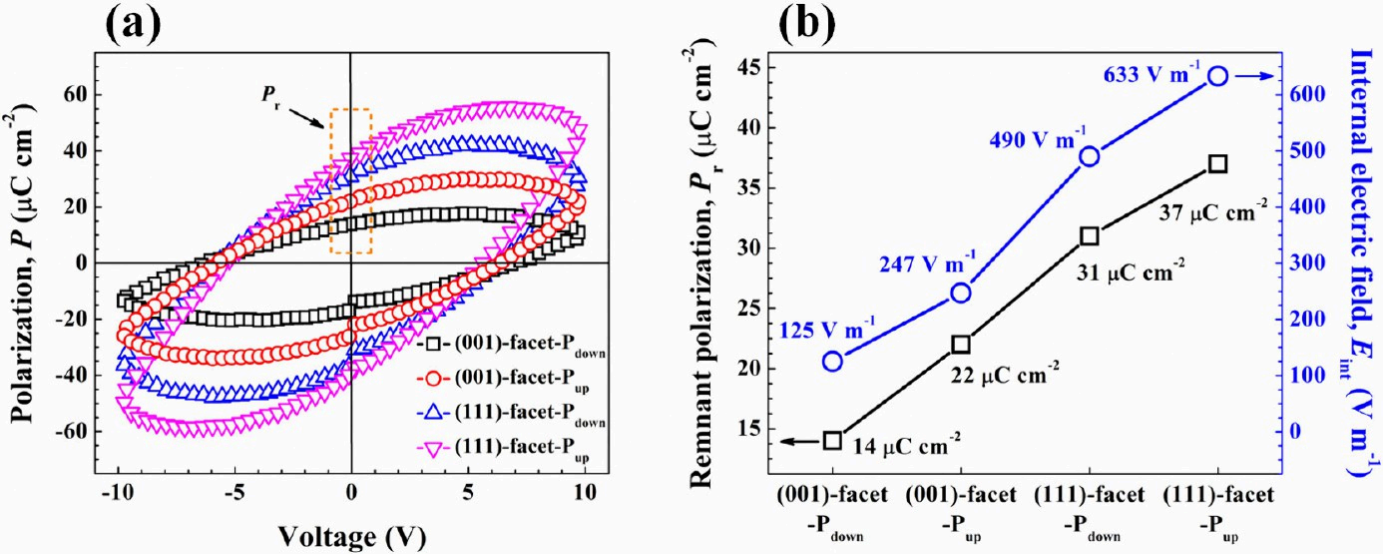
(a) Polarization–coercive voltage (*P*–*V*) hysteresis loops of (001)-facet-P_down_, (001)-facet-P_up_, (111)-facet-P_down_, and (111)-facet-P_up_. (b) Trends of obtained *P*_r_ and calculated *E*_int_ as functions of facet orientation and polarization direction.

The *E*_int_ in each BFO thin film acts as a driving force for efficient charge separation and significant reduction in electron–hole recombination. This leads to a huge charge transfer, large charge accumulation, and great band bending at the surface region. To calculate the approximate intensity of *E*_int_ for each BFO film, the following equation was employed:^45^

$$F_{s}={\left(-2V_{s}\rho /\varepsilon \varepsilon_{0}\right)}^{1/2}$$ 5

where *F*_s_ is the magnitude of *E*_int_, *V*_s_ is the surface voltage, ρ is the surface charge density, ε is the dielectric constant of BFO film,^46^ and ε_0_ represents the permittivity of free space. As depicted in Figure 6b, the order of calculated *E*_int_ magnitude from the largest to the smallest is as follows: (111)-facet-P_up_ (633 V m^–1^) > (111)-facet-P_down_ (490 V m^–1^) > (001)-facet-P_up_ (274 V m^–1^) > (001)-facet-P_down_ (125 V m^–1^), which is in excellent agreement with the trend of ferroelectric polarization strength. Among them, the *E*_int_ intensity of (111)-facet-P_up_ is particularly greater than that of (001)-facet-P_down_ by ∼5.1-fold. Hence, the robust *E*_int_ in the (111)-facet-P_up_ is expected to facilitate effective charge separation while minimizing the electron–hole recombination rate, thereby enhancing charge transfer efficiency in photoactivities.

### 3.3 Dynamics of Photogenerated Charge Carriers

KPFM was utilized to reveal the surface charge distributions for all of the BFO samples. The KPFM image of the (111)-facet-P_up_ (Figure 7a) illustrates a series of well-defined alternating bright and dark stripes, representing the surface potential (SP) measured in dark and under 405 nm illumination, respectively. The KPFM mappings for (001)-facet-P_down_, (001)-facet-P_up_, and (111)-facet-P_down_ are presented in Figure S6. In addition, Figure 7b exhibits the time evolutions of mean SP profiles for all of the BFO films. It is worth noting that the (001)-facet-P_down_ and (111)-facet-P_down_ reveal a negative SP response in the dark, while the (001)-facet-P_up_ and (111)-facet-P_up_ demonstrate a positive SP, indicating that the ferroelectric polarization direction contributes to the pristine surface charge state. Upon illumination, each BFO thin film shows a negative shift in SP evidently. The results highlight the important role of SP shifting in the charge dynamics of BFO thin films under illumination. The observed negative shift in SP indicates the accumulation of photogenerated electrons, indicating that upon photoexcitation, electron–hole pairs are effectively separated, and electrons migrate toward the surface. Besides, the surface potential difference (SPD) was used to quantify the number of photogenerated charge accumulations at the surface region, as presented in Figure 7c. Among all the BFO thin films, (111)-facet-P_up_ notably possesses the largest SPD result of −0.15 V, which surpasses (111)-facet-P_down_ (−0.11 V), (001)-facet-P_up_ (−0.08 V), and (001)-facet-P_down_ (−0.06 V). The (111)-facet-P_up_ demonstrates the highest SPD, which significantly exceeds the SPD values for the other facets. This implies that this configuration is particularly effective at accumulating a photogenerated charge at the surface. The superior charge congregation implies that stronger *E*_int_ enhances charge separation and inhibits charge recombination. On the other hand, the lower SPD in the BFO (001)-facet indicates that its weaker *E*_int_ leads to smaller charge accumulation and potentially higher electron–hole recombination rates.

**Figure 7:**
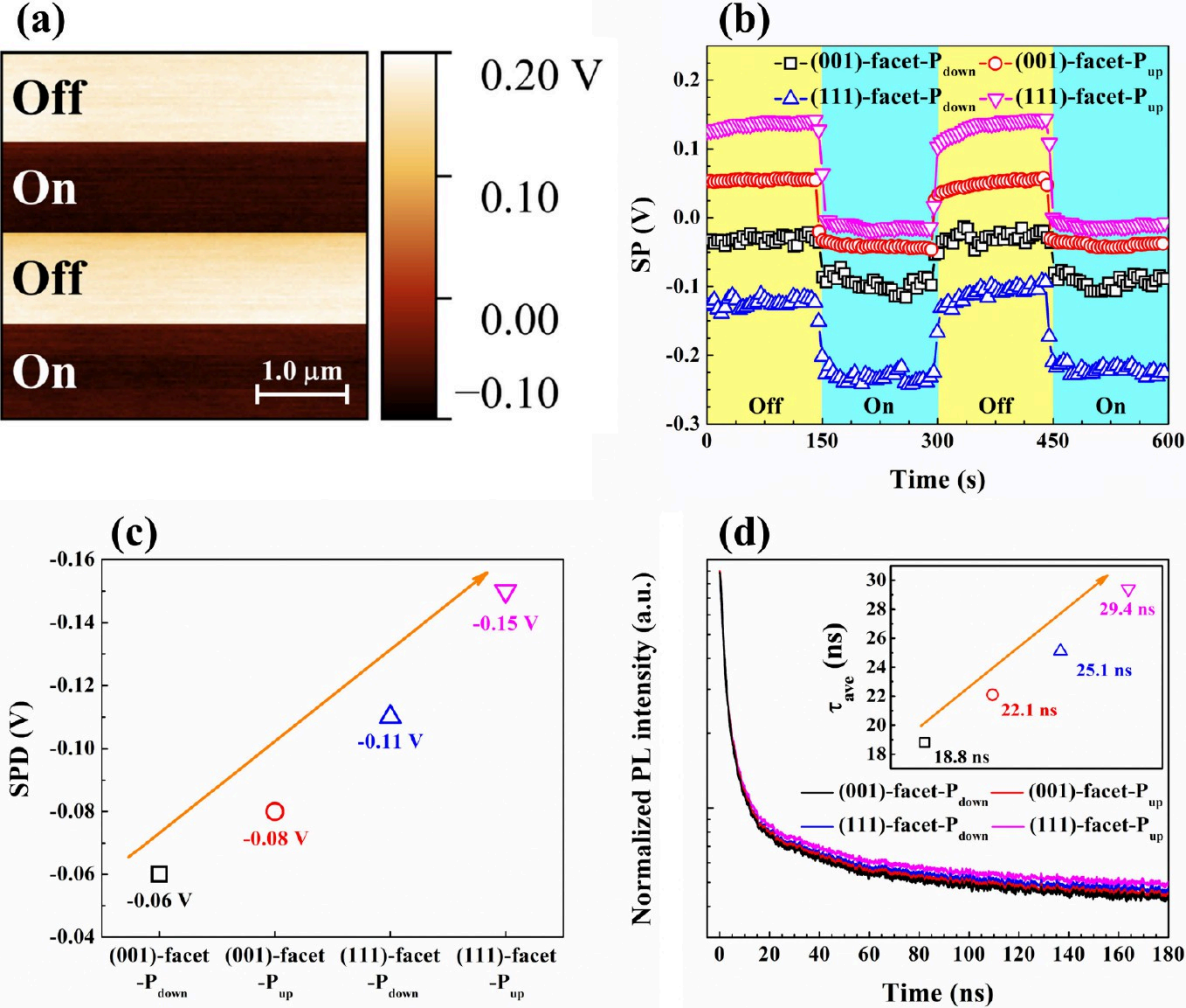
(a) KPFM image of (111)-facet-P_up_ under periodic 405 nm illumination. (b) Time evolutions of mean SP profiles for (001)-facet-P_down_, (001)-facet-P_up_, (111)-facet-P_down_, and (111)-facet-P_up_ in the dark (Off) and under light (On). (c) Trend of mean SPD as a function of facet orientation and polarization direction. (d) TRPL decay curves of corresponding samples with an inset of the τ_ave_ trend.

TRPL was employed to further explore the lifetime of the photoinduced charges for each BFO thin film. As exhibited in Figure 7d, the TRPL decay curves of (001)-facet-P_down_, (001)-facet-P_up_, (111)-facet-P_down_, and (111)-facet-P_up_ were fitted using a tri-exponential kinetic model to achieve higher fitting accuracy because this model provides a better representation of the charge decay pathway compared to the single- or bi-exponential model.^47^ The tri-exponential model can be described as follows:^47^

6 $$I\left(t\right)=A_{1}\exp\left(-t/\tau_{1}\right)+A_{2}\exp\left(-t/\tau_{2}\right)+A_{3}\exp\left(-t/\tau_{3}\right)$$

where the exponential components of τ_1_, τ_2_, and τ_3_ in the model represent the short, moderate, and long fluorescent lifetimes, respectively, while *A*_1_, *A*_2_, and *A*_3_ denote their respective amplitudes. Moreover, the physical meanings of τ_1_, τ_2_, and τ_3_ are provided in Table S2. Furthermore, the average lifetime (τ_ave_) was computed using the following equation:^47^

7 $$\tau_{\mathrm{ave}}=\left(A_{1}\tau_{1}+A_{2}\tau_{2}+A_{3}\tau_{3}\right)/\left(A_{1}+A_{2}+A_{3}\right)$$

The computed τ_ave_ results (inset of Figure 7d) demonstrate that the (111)-facet-P_up_ has the longest τ_ave_ of 29.4 ns compared to those of the (111)-facet-P_down_ (25.1 ns), (001)-facet-P_up_ (22.1 ns), and (001)-facet-P_down_ (18.8 ns). The observed results further demonstrate the role of different crystal facets in influencing the charge dynamics in BFO films. The longest τ_ave_ of 29.4 ns in the (111)-facet-P_up_ suggests that this configuration provides favorable conditions for charge carrier retention. For instance, electron–hole pairs recombine more slowly compared to the other facets. On the other hand, the shortest τ_ave_ value of 18.8 ns in the (001)-facet-P_down_ implies a faster recombination, suggesting a lower charge transport efficiency. Meanwhile, the PL spectra expressed in Figure S7 reveal a gradual decrease in PL intensity in the order: (001)-facet-P_down_ > (001)-facet-P_up_ > (111)-facet-P_down_ > (111)-facet-P_up_. The (111)-facet-P_up_ exhibits the lowest PL emission, which aligns with the observation that it suppresses electron–hole recombination more effectively. A lower PL intensity correlates with reduced recombination losses, implying that this facet retains charge carriers for a longer duration and potentially enhances charge extraction efficiency.

### 3.4 Performance of PEC Water Splitting

The energy band edge positions of all of the BFO samples are essential to determine the water redox behaviors. To begin with, the flat band potential (*E*_fb_) is acquired by extrapolating the Mott–Schottky (MS) slope toward the potential axis. As shown in Figure S8, the *E*_fb_ results of (001)-facet-P_down_, (001)-facet-P_up_, (111)-facet-P_down_, and (111)-facet-P_up_ are thus estimated to be 0.02, 0.04, 0.06, and 0.10 V vs Ag/AgCl, respectively. Meanwhile, the negative MS slope for each BFO film confirms the p-type semiconductor characteristic, which is in excellent agreement with the XPS investigation (Figure S3). To build a standardized reference for energy conversion, the *E*_fb_ is converted to a normal hydrogen electrode potential (*E*_NHE_) using the following equation:^48^

8 $$E_{\mathrm{NHE}}=E_{\mathrm{Ag}\text{/}\mathrm{AgCl}}+0.1967\;V$$

where *E*_Ag/AgCl_ represents the potential vs Ag/AgCl and 0.1967 V corresponds to the potential of the Ag/AgCl electrode saturated with 3.0 M KCl. Therefore, the respective *E*_fb_ levels are calculated to be 0.22, 0.24, 0.26, and 0.30 V vs NHE for the (001)-facet-P_down_, (001)-facet-P_up_, (111)-facet-P_down_, and (111)-facet-P_up_. In general, the valence band maximum (VBM) of p-type semiconductors is 0.2 V more electropositive than the *E*_fb_;^49^ their VBM are further estimated to be 0.42, 0.44, 0.46, and 0.50 V vs NHE, respectively. By combining the derived *E*_g_ values, the positions of the VBM and conduction band minimum (CBM) for all of the BFO films are illustrated in Figure 8a. Evidently, the CBM level of each BFO sample is positioned well above the water reduction potential (−0.41 V, H^+^/H_2_), indicating that the prepared BFO thin films are appropriate for photocathodes in the PEC hydrogen evolution reaction (HER).

**Figure 8:**
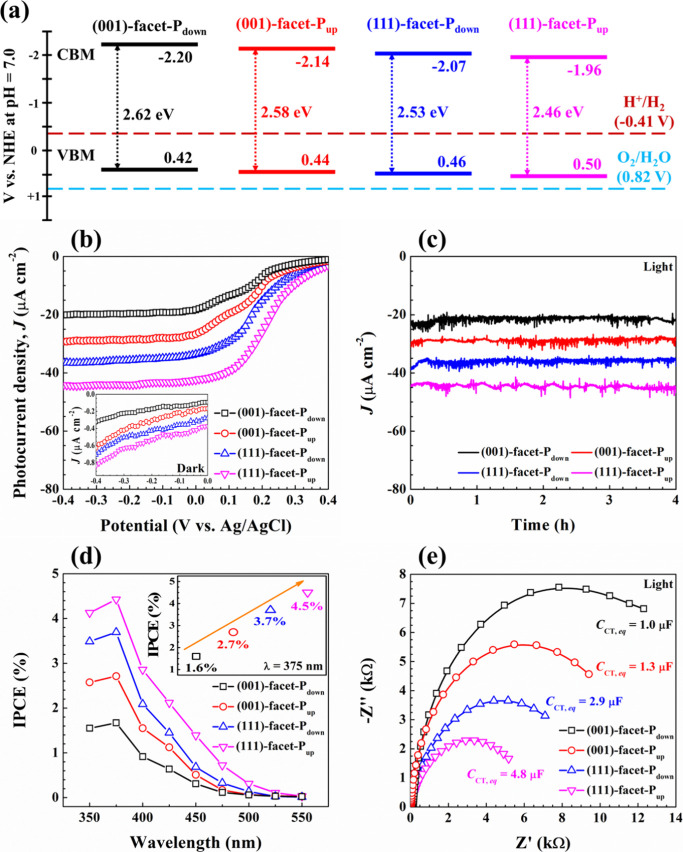
(a) Schematic diagrams of band energy levels for (001)-facet-P_down_, (001)-facet-P_up_, (111)-facet-P_down_, and (111)-facet-P_up_ in 0.5 M Na_2_SO_4_ electrolyte (pH = 7.0). PEC characterizations of the corresponding samples measured under AM1.5G simulated solar light. (b) LSV curves. The inset shows the LSV examined in dark. (c) 4 h CA stability. (d) IPCE spectra as a function of wavelength. The inset exhibits the collected IPCE values at the λ = 375 nm. (e) EIS Nyquist plots measured under light. (c), (d), and (e) were conducted at 0 V vs. Ag/AgCl.

To probe the effect of ferroelectric polarization-induced electric field (*E*_int_) strength on PEC performance, the LSV, 4 h CA stability, IPCE spectra, and EIS Nyquist plot were evaluated. Under AM1.5G illumination, all the p-type BFO photoelectrodes reveal the photocathodic behavior in the applied potential range from −0.4 to 0 V vs. Ag/AgCl (Figure 8b). The photocurrent density is ∼2-order greater than the current response measured in the dark (inset of Figure 8b), indicating spatial electron–hole separation and significant charge transfer across the BFO/electrolyte interface driven by light excitation and *E*_int_. A stronger magnitude of *E*_int_ mitigates charge recombination effectively, thus amplifying the surface charge density and surface band bending at the BFO/electrolyte interface. The CA examination (Figure 8c) demonstrates that all the BFO samples possess stable photocurrent densities throughout the entire 4 h illumination. Meanwhile, the (111)-facet-P_up_ has the highest photocurrent density of −45 μA cm^–2^, which surpasses the (111)-facet-P_down_ (−37 μA cm^–2^), (111)-facet-P_up_ (−28 μA cm^–2^), and (001)-facet-P_down_ (−21 μA cm^–2^). Besides, the wavelength-dependent IPCE spectra (Figure 8d) reveal a significant increase in photoactivity across all the BFO samples in the wavelength (λ) range of 350–500 nm. According to the IPCE measurements, the maximum IPCE response is observed at λ = 375 nm. As exhibited in the inset of Figure 8d, the (111)-facet-P_up_ demonstrates the largest IPCE result of 4.5%, followed by (111)-facet-P_down_ (3.7%), (001)-facet-P_up_ (2.7%), and (001)-facet-P_down_ (1.6%). Meanwhile, EIS Nyquist plots were conducted to examine the charge transfer efficiency across the BFO/electrolyte interface. The equivalent circuit model (Figure S9) was employed to gain the well-fitted impedance results, where R_CT_ represents the charge transfer resistance and CPE_CT_ is the constant phase element of charge transfer. The fitted R_CT_ and CPE_CT_ results for all of the BFO samples are provided in Table S1. Notably, the (111)-facet-P_up_ gives rise to the smallest semi-circle radius, indicative of the lowest R_CT_ with the most efficient interfacial charge transfer, as shown in Figure 8e. Additionally, the equivalent capacitance of charge transfer (*C*_CT, eq_) was calculated by the following equation:^50^

$$C_{CT,\;eq}=\left({\left(Y_{0}R_{p}\right)}^{1/n}/R_{p}\right)\sin\left(n\pi /2\right)$$ 9

where *R*_*p*_ is R_CT_, *Y*_0_ is CPE_CT_, and *n* is the exponent (0 < *n* < 1) of CPE_CT_. It should be noted that *C*_CT, eq_ can be used to quantify the density of charge carrier transport across the BFO/electrolyte interface. As presented in Figure 8e, the order of calculated *C*_CT, eq_ from the largest to the smallest is as follows: (111)-facet-P_up_ (4.8 μF) > (111)-facet-P_down_ (2.9 μF) > (001)-facet-P_up_ (1.3 μF) > (001)-facet-P_down_ (1.0 μF). The highest *C*_CT, eq_ in the (111)-facet-P_up_ correlates with increased photocurrent densities and IPCE. This facet optimally facilitates charge separation and transport, reducing charge recombination losses and improving overall PEC efficiency. In particular, the stronger *E*_int_ in (111)-facet-P_up_ drives efficient electron–hole separation. This leads to lower recombination rates, allowing more charge carriers to be transferred across the BFO/electrolyte interface.

### 3.5 DFT Calculations

DFT calculations were carried out to discover the role of ferroelectric polarization in tuning surface electronic properties of (001)-facet-P_down_, (001)-facet-P_up_, (111)-facet-P_down_, and (111)-facet-P_up_, as shown in Figure 9. To begin with, the polarization direction is found to have a significant effect on changing the edge positions of the valence band (VB) and conduction band (CB). In specific, the VB edges of (001)-facet-P_down_ and (111)-facet-P_down_ are closer to the Fermi level (*E*_F_), whereas the CB edges of (001)-facet-P_up_ and (111)-facet-P_up_ nearly overlap with the *E*_F_. The difference in the energy band edge position is ascribed to the transport dynamics of surface electrons and holes. In particular, the *E*_int_ of P_down_ facilitates more surface hole migration, while the *E*_int_ of P_up_ promotes more surface electron transfer.^17^ These findings indicate that ferroelectric polarization can actively modulate the surface electronic states, imparting the charge transport mechanism. The finding agrees with the KPFM examinations which shows the effect of P_up_ (P_down_) leads to the positive (negative) surface charge state in the dark condition (Figure 7b). In addition, the DFT calculations demonstrate that (111)-facet-P_up_ (Figure 9d) exhibits a higher peak intensity in the entire VB and CB regions compared to the (001)-facet-P_up_ (Figure 9b). This indicates that (111)-facet-P_up_ enables stronger electronic interactions compared to the (001)-facet-P_up_. A similar trend is found when comparing (111)-facet-P_down_ (Figure 9c) with (001)-facet-P_down_ (Figure 9a), suggesting that the (111)-facet generally supports enhanced surface electronic activity. Therefore, the order of the surface charge intensity from the greatest to the lowest is as follows: (111)-facet-P_up_ > (111)-facet-P_down_ > (001)-facet-P_up_ > (001)-facet-P_down_. It should be noted that the presence of *E*_int_ strength acts as the key factors for determining the amount of charge carrier transfer toward the BFO surface. Based on this, an enhanced density of surface charges in the (111)-facet-P_up_ can be attributed to its larger *E*_int_ strength, which excellently supports the results in the ferroelectric measurements (Figure 6).

**Figure 9:**
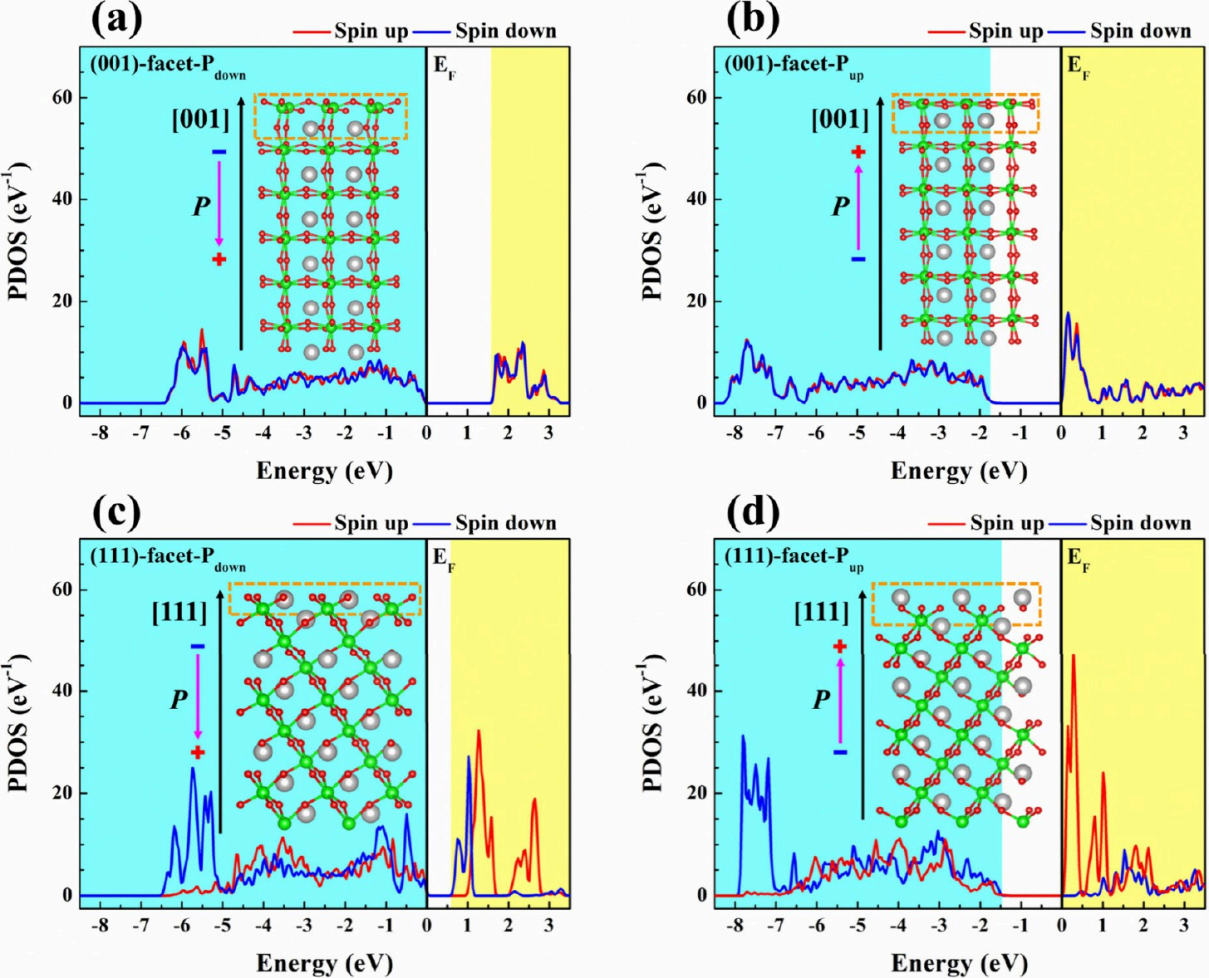
Calculated PDOS of (a) (001)-facet-P_down_, (b) (001)-facet-P_up_, (c) (111)-facet-P_down_, and (d) (111)-facet-P_up_, extracted from the corresponding top layers (orange dashed boxes) of the inset slab models. The gray, green, and red spheres in the slab models indicate the Bi, Fe, and O atoms, respectively. The black arrow near the slab model represents the crystallographic orientation. The blue (yellow) region denotes the valence (conduction) band. The black solid line at 0 eV is the Fermi level (*E*_F_).

To gain further insight into the influence of ferroelectric behaviors on charge transfer efficiency in the BFO samples, DFT calculations of electron local potentials were used to estimate the work functions (Φ) of (001)-facet-P_down_, (001)-facet-P_up_, (111)-facet-P_down_, and (111)-facet-P_up_, as illustrated in Figure 10. It should be noted that a dipole correction perpendicular to all the BFO surfaces is applied to ensure absence of net charges which could affect the potential at the vacuum level. It is essential to recognize that the effectiveness of charge migration in BFO can be impacted by the ferroelectric polarization strength. In other words, the performance of *E*_int_ could directly influence the Φ value. The theoretical computations reveal that (111)-facet-P_up_ possesses the lowest Φ of 1.74 eV compared to (111)-facet-P_down_ (1.93 eV), (001)-facet-P_up_ (2.09 eV), and (001)-facet-P_down_ (2.24 eV). A comparative analysis between the BFO (111)-facets and BFO (001)-facets indicates that the former has the smaller Φ. In addition, the P_up_ state in BFO enables a reduction in Φ compared to the P_down_ condition.

**Figure 10:**
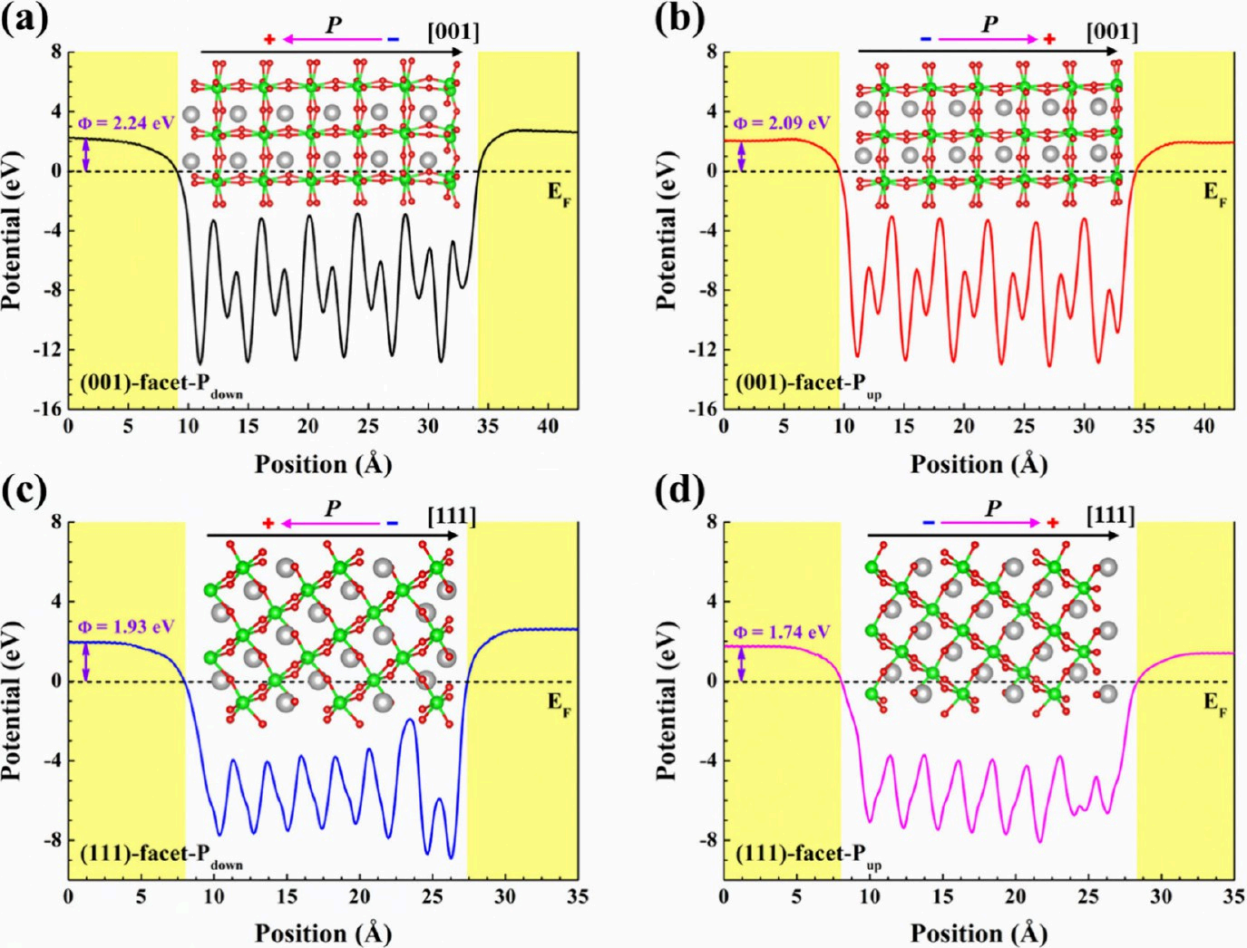
Calculated electron local potential profiles and work functions (Φ) of (a) (001)-facet-P_down_, (b) (001)-facet-P_up_, (c) (111)-facet-P_down_, and (d) (111)-facet-P_up_. The yellow region denotes the vacuum region. The black dashed line at 0 eV is the Fermi level (*E*_F_).

The work function (Φ) of a material, which defines the minimum energy required to extract an electron from a material into a vacuum, plays a crucial role in governing interfacial charge transfer processes. In BFO thin films, ferroelectric polarization provides an effective means of tuning the surface energetics and modulating the effective work function.^65–67^ Upward polarization induces downward band bending at the BFO surface, effectively lowering the Φ and reducing the energy barrier for electron transfer to the electrolyte. This facilitates the hydrogen evolution reaction in PEC water reduction by enhancing charge injection and suppressing recombination losses. A reduced work function not only promotes more efficient electron extraction but also minimizes charge accumulation at the interface, thereby improving charge separation and increasing photocurrent. Compared to the (001)-facet, the (111)-facet exhibits a lower surface defect density, further minimizing recombination centers.^68^ In addition, the (111)-facet can support higher charge mobility due to its more symmetric atomic configuration, which reduces electron–phonon scattering and improves orbital overlap between Fe 3d and O 2p states, thereby enhancing charge delocalization.^69^ Meanwhile, the alignment of ferroelectric polarization along the out-of-plane [111] direction in the (111)-facet induces huge downward band bending at the surface, which effectively reduces the Φ and lowers the energy barrier for electron transportation. This large polarization-induced internal electric field consequently gives rise to efficient separation and migration of photogenerated charges. Overall, these advantages in the (111)-facet-P_up_ enable it to possess the lowest electrical leakage (Figure 5a), the largest ferroelectricity (Figure 6), the most effective photoexcited charge transport (Figure 7c), and the longest charge carrier lifetime (Figure 7d), thereby significantly improving its electron–hole separation and inhibiting charge recombination in the PEC water reduction.

### 3.6 Proposed Mechanisms of PEC Water Splitting

The mechanisms of PEC water splitting for (001)-facet-P_down_, (001)-facet-P_up_, (111)-facet-P_down_, and (111)-facet-P_up_ are schematically expressed in Figure 11. The ferroelectric polarization-induced internal electric field (*E*_int_) coupled with surface band bending is found to be dependent on the choice of facet orientations and polarization directions, thereby manipulating the efficiency of photoexcited charge carriers in PEC water reduction. Since the *E*_int_ of P_down_ is opposite with the electric field generated by the pure p-type BFO,^11,56^ the opposing electric fields create a relatively small downward band bending at the BFO/electrolyte interface. Thus, the driving force for photogenerated electron transfer is weak. In contrast, the *E*_int_ of P_up_ is in the same direction as the field of p-type BFO.^11,56^ This leads to large downward band bending, which provides a strong driving force for electron migration across the BFO/electrolyte interface. In addition, a larger *E*_int_ strength along with a smaller leakage current can contribute to huge electron–hole separation and therefore increases the lifetime of photoexcited charges. Among all the BFO photocathodes, the (111)-facet-P_up_ (Figure 11d) particularly stands out owing to its optimized ferroelectric polarization, electrical property, and charge dynamics, resulting in an outstanding photoexcited electron transport and large downward band bending in the PEC water reduction.

**Figure 11:**
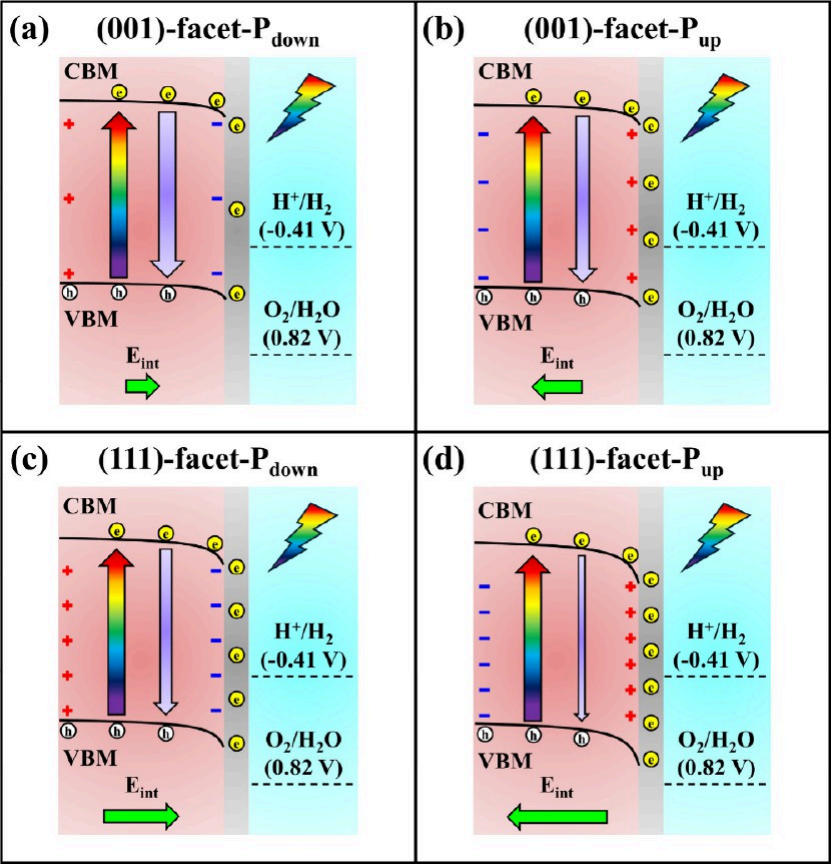
Schematic illustrations of PEC water splitting mechanisms for (a) (001)-facet-P_down_, (b) (001)-facet-P_up_, (c) (111)-facet-P_down_, and (d) (111)-facet-P_up_. The length of the green arrow indicates the strength of ferroelectric polarization-induced internal electric field (*E*_int_). The width of purple arrow suggests the recombination rate of photoexcited electron–hole pairs.

## 4 Conclusions

In summary, this work evaluated the influence of crystal facet orientation and spontaneous polarization direction on electrical leakage, ferroelectric polarization-induced internal electric field (*E*_int_), charge dynamics, and photoelectrochemical (PEC) water reduction of p-type BFO thin film heterostructures. Both experimental results and DFT calculations demonstrate that the facet orientation and polarization direction play a vital role in modulating the leakage current and *E*_int_ magnitude, thereby tuning the charge migration property and efficiency. Among all the BFO samples, the (111)-facet-P_up_ exhibits the most efficient charge carrier transfer, lowering the leakage current density by ∼1-order and subsequently increasing the *E*_int_ strength by ∼5.1-fold. These improved electrical and ferroelectric characterizations effectively facilitate the separation of photogenerated electron–hole pairs and suppress the charge recombination, which prolongs charge carrier lifetime upon illumination. Consequently, the (111)-facet-P_up_ possesses a ∼2.8-fold enhancement in incident photon-to-current efficiency (IPCE) (at λ = 375 nm) during the PEC water reduction activity. Meanwhile, a ∼4.8-fold increase in photoexcited charge transfer density substantially amplifies downward band bending degree at the BFO/electrolyte interface. This study shows the importance of synergistic effect of facet orientation and polarization direction in developing effective ferroelectric-based photoelectrodes, thus delivering a generic approach for rational design of ferroelectric materials to enhance solar-to-hydrogen conversion efficiency in PEC water splitting.

## Supplementary Material



## Notes

Wide XRD patterns, FESEM surface morphologies, XPS valence band spectra of BFO/LSMO (111), PFM phase hysteresis loops of BFO/SRO (111) and BFO/LSMO (111), leakage current mechanism of PF model, KPFM images under periodic illumination, PL spectra, Mott–Schottky plots, equivalent circuit model of EIS, energy band diagrams with estimated work functions, energy band diagrams under V vs NHE and V vs vacuum, fitted results in the EIS measurement, and physical meanings of τ_1_, τ_2_, and τ_3_ (PDF)
