# Supplementary material for: Synergistic Role of Facet-Engineered Surface and Ferroelectric Polarization in Photoelectrochemical Water Reduction over Pure BiFeO3 Thin Film
Source: ACS Appl Mater Interfaces. 2025 Aug 1;17(32):46339–52. doi: 10.1021/acsami.5c09048 (PMC12356542; doi:10.1021/acsami.5c09048)
Supplement: Supplementary file 1 [file am5c09048_si_001.pdf]

Supporting Information

Synergistic Role of Facet-Engineered Surface and Ferroelectric Polarization in Photoelectrochemical Water Reduction over Pure $BiFeO_3$ Thin Film

Ming-Wei Chu,[a] Yun-Wen Chen,[b] Khian Hooi Chew,[c] Narong Chanlek,[d] Cheng-Sao Chen,[e] Chang Fu Dee,[f] and Wei Sea Chang,[a,g]*

[a] Mechanical Engineering Discipline, School of Engineering, Monash University Malaysia, Bandar Sunway, Selangor, 47500, Malaysia

[b] Graduate Institute of Electronics Engineering, and Department of Electrical Engineering, National Taiwan University, Taipei 10617, Taiwan

[c] Key Laboratory of Optical Field Manipulation of Zhejiang Province, Department of Physics, Zhejiang Sci-Tech University, Hangzhou 310018, China; Zhejiang Expo New Materials Co. Ltd., Wenzhou 325802, China

[d] Synchrotron Light Research Institute (Public Organization), 111 University Avenue, Muang, Nakhon Ratchasima 30000, Thailand

[e] Department of Mechanical Engineering, Hwa Hsia University of Technology, New Taipei City 23567, Taiwan

[f] Institute of Microengineering and Nanoelectronics (IMEN), Universiti Kebangsaan Malaysia (UKM), 43600 Bangi, Selangor, Malaysia

[g] Department of Materials Science and Engineering, National Yang Ming Chiao Tung University, Hsinchu 30010, Taiwan

*Corresponding author

*Email: weiseachang@nycu.edu.tw

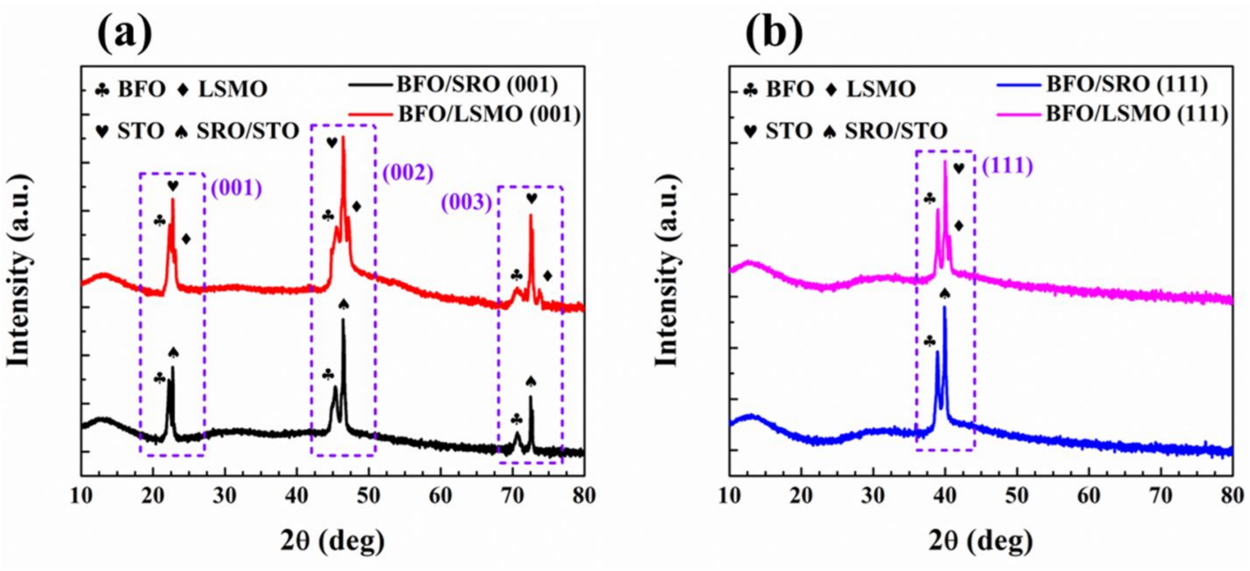

Figure S1. XRD patterns of BFO/SRO and BFO/LSMO grown on (a) STO (001) and (b) STO (111) substrates ranging from 10° to 80°.

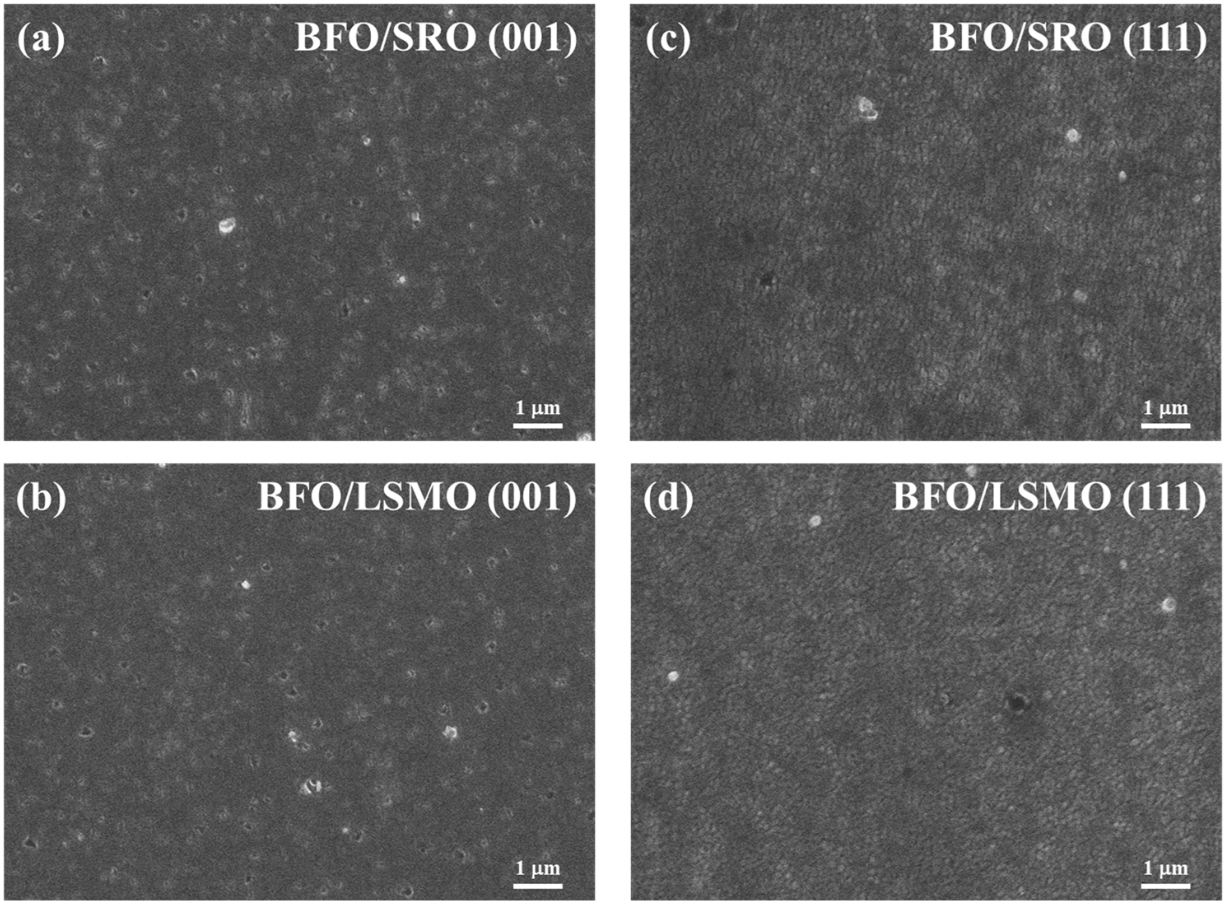

Figure S2. FESEM surface morphologies of (a) BFO/SRO (001), (b) BFO/LSMO (001), (c) BFO/SRO (111), and (d) BFO/LSMO (111).

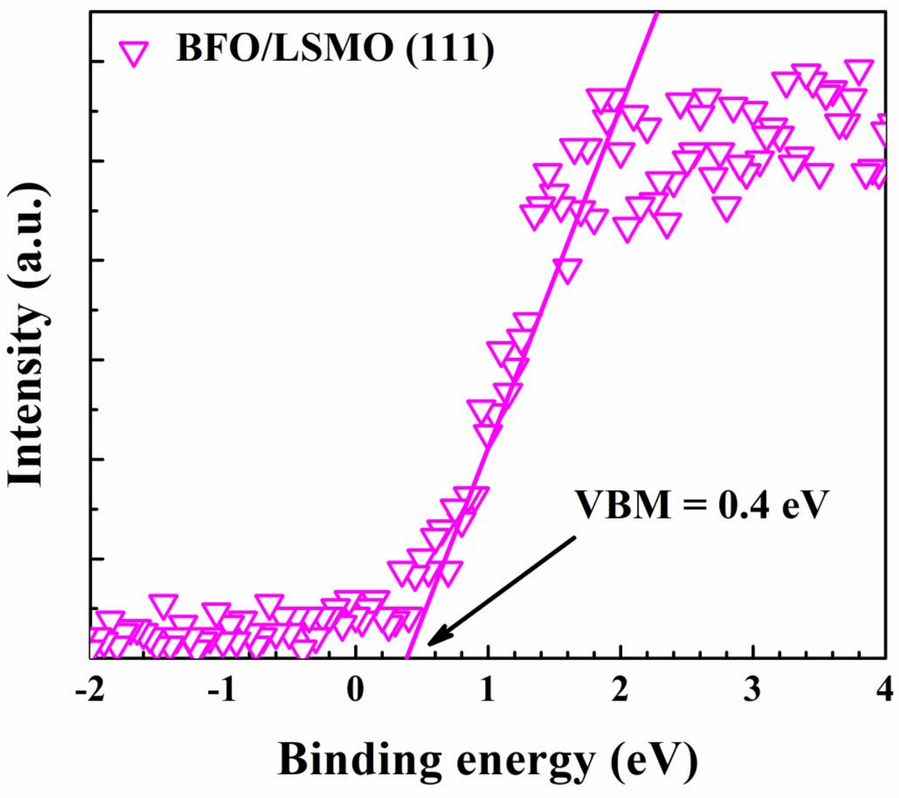

Figure S3. XPS valence band spectra of BFO/LSMO (111).

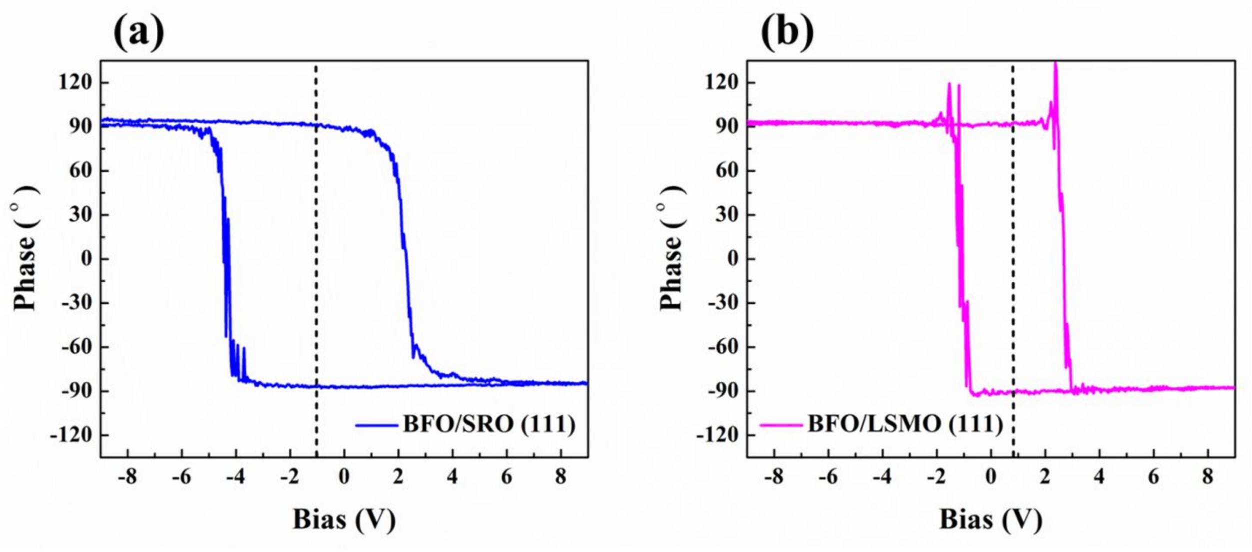

Figure S4. PFM phase hysteresis loops of (a) BFO/SRO (111) and (b) BFO/LSMO (111).

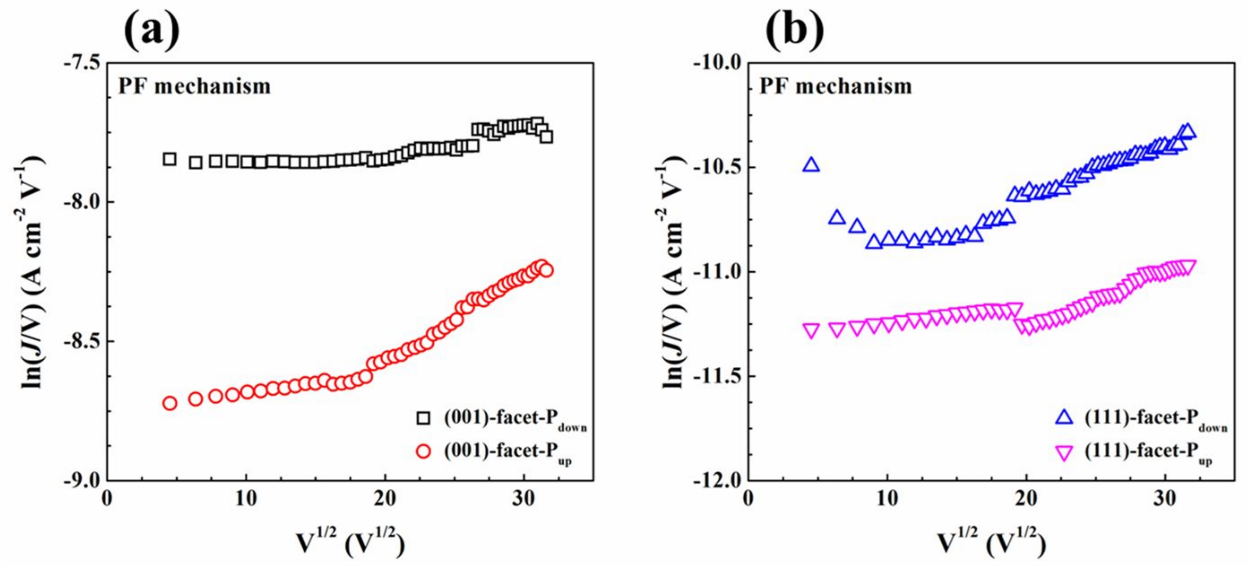

Figure S5. Leakage current mechanism of PF model for (a) (001)-facet-P$_{down}$ and (001)-facet-P$_{up}$ and (b) (111)-facet-P$_{down}$ and (111)-facet-P$_{up}$.

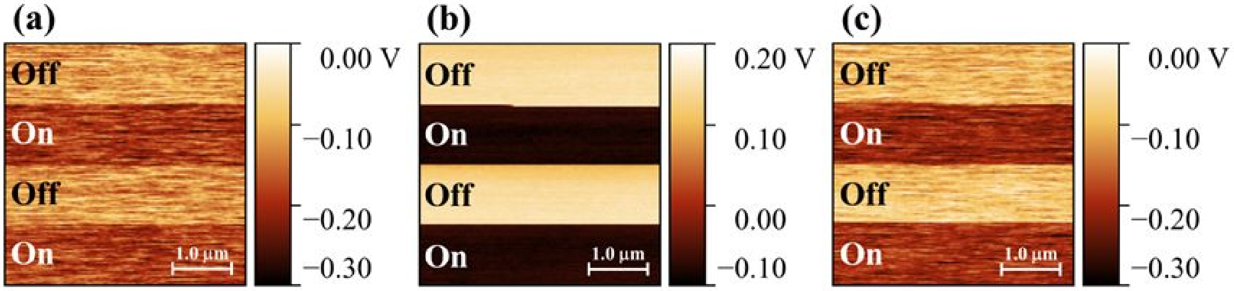

Figure S6. KPFM images of (a) (001)-facet-P$_{down}$, (b) (001)-facet-P$_{up}$, and (c) (111)-facet-P$_{down}$ under periodic illumination.

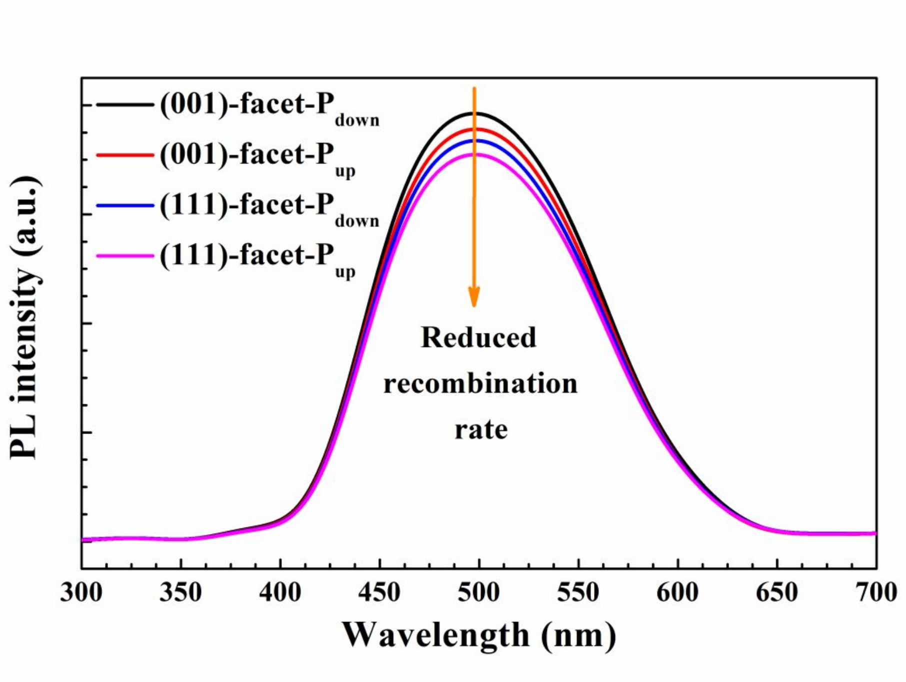

Figure S7. PL spectra of (001)-facet-P_{down}, (001)-facet-P_{up}, (111)-facet-P_{down}, and (111)-facet-P_{up}.

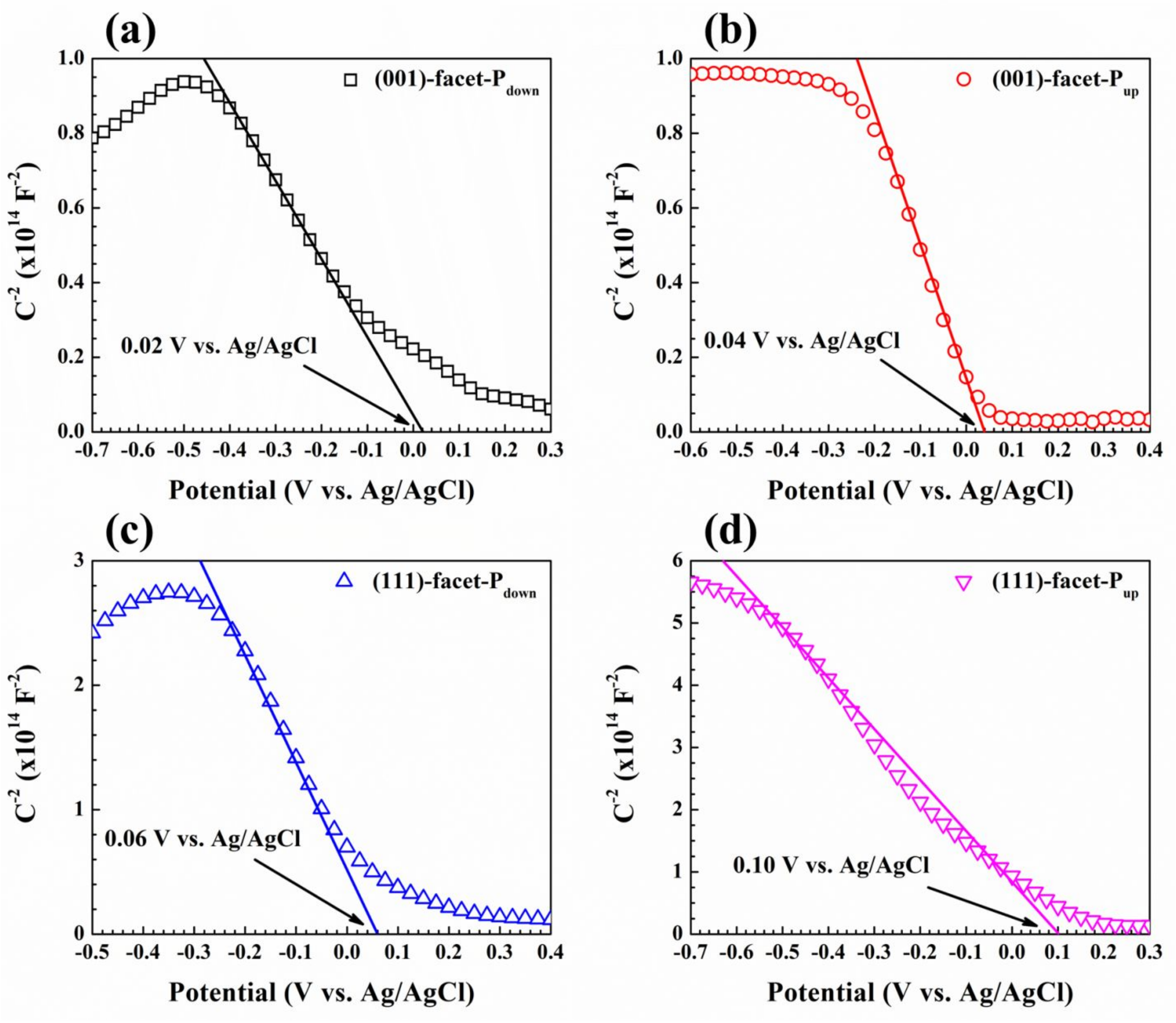

Figure S8. Mott-Schottky plots of (a) (001)-facet-P_{down}, (b) (001)-facet-P_{up}, (c) (111)-facet-P_{down}, and (d) (111)-facet-P_{up} in 0.5 M Na_2SO_4 electrolyte (pH = 7.0).

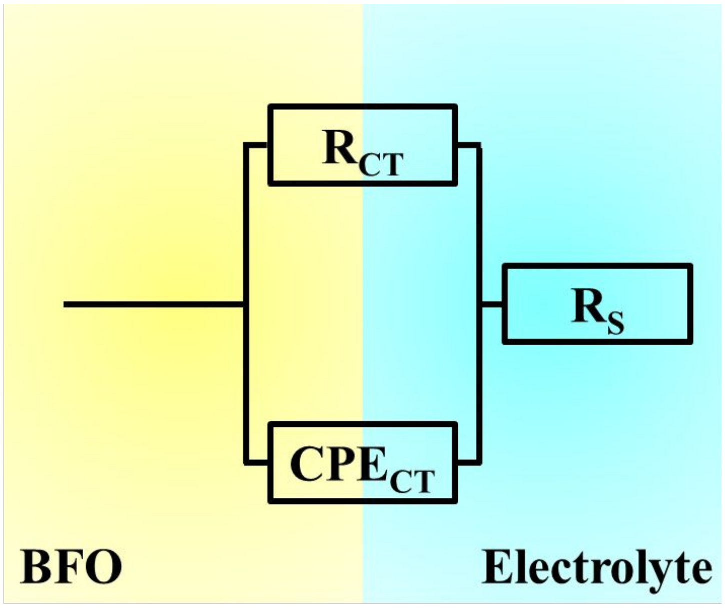

Figure S9. Equivalent circuit model of all the BFO samples.

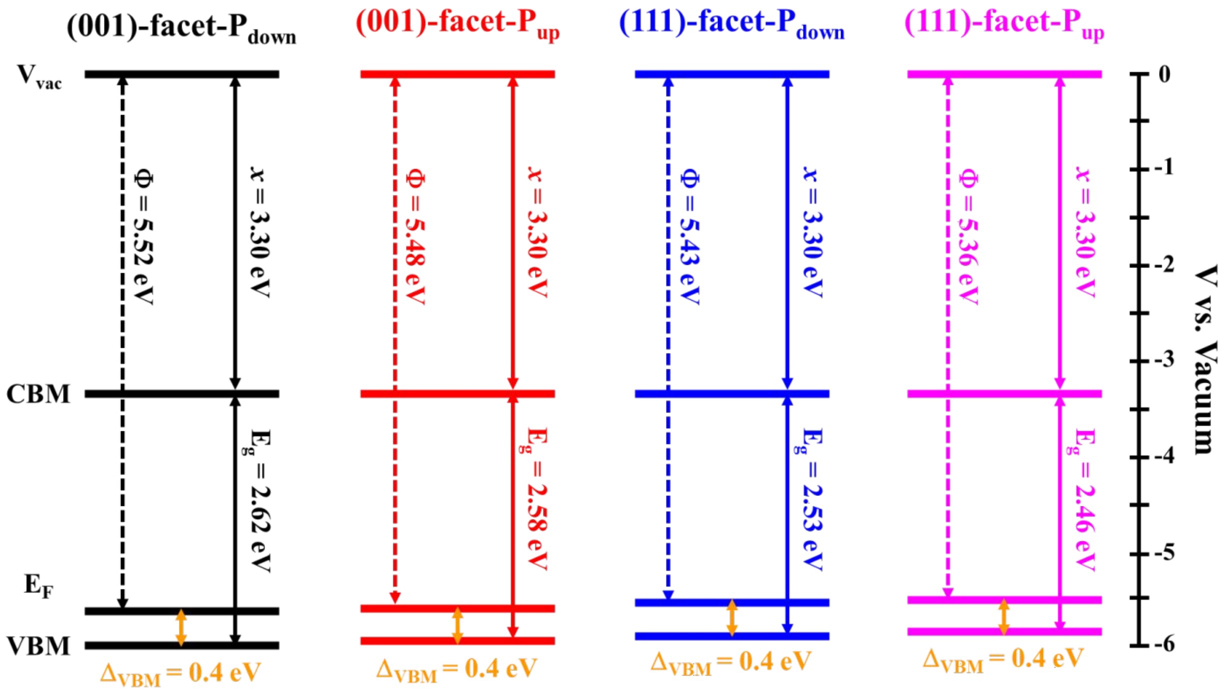

Figure S10. Energy band diagrams with estimated work functions (Φ) for (001)-facet-P_{down}, (001)-facet-P_{up}, (111)-facet-P_{down}, and (111)-facet-P_{up} under V vs. Vacuum. The dash line indicates the Φ.

The detailed Φ calculation for all the BFO samples can be described as:

$\Phi = x + (E_g - \Delta_{VBM})$

where x is the electron affinity (~3.30 eV) of BFO,[1] E_g is the bandgap energy of BFO, and Δ_{VBM} is the energy gap (~0.40 eV) between the valence band maximum (VBM) and the Fermi level (E_F).

Φ of (001)-facet-P_{down} = 3.30 eV + (2.62 eV – 0.4 eV) = 5.52 eV

Φ of (001)-facet-P_{up} = 3.30 eV + (2.58 eV – 0.4 eV) = 5.48 eV

Φ of (111)-facet-P_{down} = 3.30 eV + (2.53 eV – 0.4 eV) = 5.43 eV

Φ of (111)-facet-P_{up} = 3.30 eV + (2.46 eV – 0.4 eV) = 5.36 eV

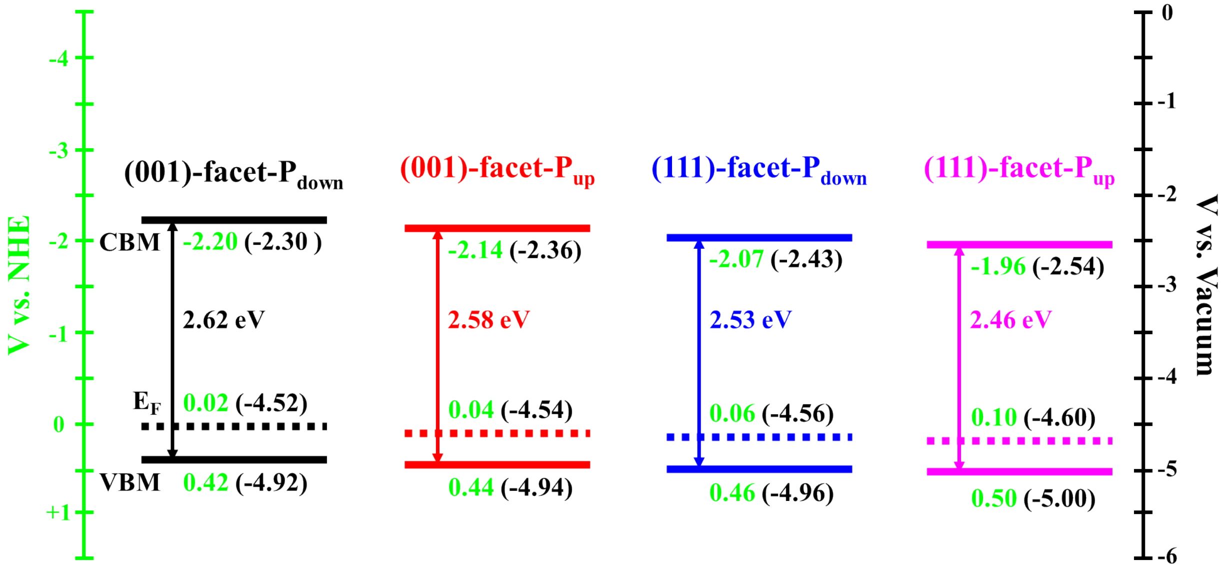

Figure S11. Energy band diagrams of (001)-facet-P_{down}, (001)-facet-P_{up}, (111)-facet-P_{down}, and (111)-facet-P_{up} under V vs. NHE (green color) and V vs. Vacuum (black color). The dash line indicates the Fermi level (E_F).

The relationship between V vs. NHE and V vs. Vacuum can be described as follows:[2]

$$V_{Vacuum} = V_{NHE} - 4.5\ V$$

where the V_{Vacuum} and V_{NHE} are the potentials referenced to vacuum and NHE, respectively. 4.5 V is the energy difference between the vacuum level and the NHE level.

Table S1. The fitted results of individual circuit elements in the EIS measurements of (001)-facet-P_{down}, (001)-facet-P_{up}, (111)-facet-P_{down}, and (111)-facet-P_{up} under illumination.

Sample	R_{CT} (kΩ)	CPE_{CT} ($S \cdot s^n$)	n
(001)-facet-P_{down}	15.6	2.51E–6	0.79
(001)-facet-P_{up}	11.2	4.02E–6	0.75
(111)-facet-P_{down}	8.8	5.57E–6	0.83
(111)-facet-P_{up}	6.8	7.37E–6	0.88

Table S2. The physical meanings of the three decay components (τ_1, τ_2, and τ_3).

Decay component	Physical meaning
τ_1	This represents ultrafast decay, typically attributed to trap-induced recombination due to shallow defects. It reflects the rapid recombination of photoexcited charges at defect sites
τ_2	This is associated with bulk recombination processes, where photogenerated charge carriers recombine within the interior of the semiconductor. It reflects intrinsic charge dynamics that are not strongly influenced by defects or interfaces
τ_3	This corresponds to the long-lived charge carriers, typically resulting from deep trap states or effective charge separation. It reflects slow recombination of photoexcited charges

References

(1) Clark, S. J.; Robertson, J. Band gap and Schottky barrier heights of multiferroic $BiFeO_3$. *Appl. Phys. Lett.* **2007**, *90,* 132903.

(2) Trasatti, S. The absolute electrode potential: an explanatory note. *Pure Appl. Chem.* **1986**, *58,* 955–966.
